# Emergence of MXene and MXene–Polymer Hybrid Membranes as Future‐ Environmental Remediation Strategies

**DOI:** 10.1002/advs.202203527

**Published:** 2022-10-31

**Authors:** Ajit Khosla, Sonu, Hafiz Taimoor Ahmed Awan, Karambir Singh, Gaurav, Rashmi Walvekar, Zhenhuan Zhao, Ajeet Kaushik, Mohammad Khalid, Vishal Chaudhary

**Affiliations:** ^1^ Department of Applied Chemistry School of Advanced Materials and Nanotechnology Xidian University Xi'an 710126 P. R. China; ^2^ School Advanced of Chemical Sciences Shoolini University of Biotechnology and Management Sciences Bajhol Solan HP 173212 India; ^3^ Graphene and Advanced 2D Materials Research Group (GAMRG) School of Engineering and Technology Sunway University No. 5 Jalan University Bandar Sunway Petaling Jaya Selangor 47500 Malaysia; ^4^ School of Physics and Material science Shoolini University of Biotechnology and Management Sciences Bajhol Solan HP 173212 India; ^5^ Department of Botany Ramjas College University of Delhi Delhi 110007 India; ^6^ Department of Chemical Engineering School of New Energy and Chemical Engineering Xiamen University Malaysia Jalan Sunsuria, Bandar Sunsuria Sepang Selangor 43900 Malaysia; ^7^ NanoBioTech Laboratory Health System Engineering Department of Environmental Engineering Florida Polytechnic University Lakeland FL 33805 USA; ^8^ School of Engineering University of Petroleum and Energy Studies (UPES) Dehradun Uttarakhand 248007 India; ^9^ Sunway Materials Smart Science and Engineering (SMS2E) Research Cluster Sunway University No. 5 Jalan Universiti Bandar Sunway Petaling Jaya Selangor 47500 Malaysia; ^10^ Research Cell and Department of Physics Bhagini Nivedita College University of Delhi New Delhi India; ^11^ SUMAN Laboratory (SUstainable Materials and Advanced Nanotechnology Lab) University of Delhi New Delhi 110072 India

**Keywords:** electromagnetic shielding, environmental remediation, gas separation, intelligent membrane, MXene–polymer hybrids, water purification

## Abstract

The continuous deterioration of the environment due to extensive industrialization and urbanization has raised the requirement to devise high‐performance environmental remediation technologies. Membrane technologies, primarily based on conventional polymers, are the most commercialized air, water, solid, and radiation‐based environmental remediation strategies. Low stability at high temperatures, swelling in organic contaminants, and poor selectivity are the fundamental issues associated with polymeric membranes restricting their scalable viability. Polymer‐metal‐carbides and nitrides (MXenes) hybrid membranes possess remarkable physicochemical attributes, including strong mechanical endurance, high mechanical flexibility, superior adsorptive behavior, and selective permeability, due to multi‐interactions between polymers and MXene's surface functionalities. This review articulates the state‐of‐the‐art MXene–polymer hybrid membranes, emphasizing its fabrication routes, enhanced physicochemical properties, and improved adsorptive behavior. It comprehensively summarizes the utilization of MXene–polymer hybrid membranes for environmental remediation applications, including water purification, desalination, ion‐separation, gas separation and detection, containment adsorption, and electromagnetic and nuclear radiation shielding. Furthermore, the review highlights the associated bottlenecks of MXene–Polymer hybrid‐membranes and its possible alternate solutions to meet industrial requirements. Discussed are opportunities and prospects related to MXene–polymer membrane to devise intelligent and next‐generation environmental remediation strategies with the integration of modern age technologies of internet‐of‐things, artificial intelligence, machine‐learning, 5G‐communication and cloud‐computing are elucidated.

## Graphical abstract

2D MXenes–polymer hybrid based membranes possess colossal latent to transform the environmental remediation technologies owing to their remarkable physicochemical and sustainable attributes, facilitating attainment of smart, flexible, high adsorption efficacies, cost‐effective, energy‐efficient, compact, portable, point‐of‐solution, scavenging, self‐powered, multifunctional, integrated on a single membrane supported by advanced technologies including machine learning, artificial intelligence, 5G wireless communication, cloud computing, and internet‐of‐things.

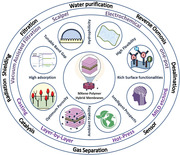

## 1 Introduction: Emergence of MXene–Polymer Hybrid Membranes for Environmental Remediation

In this era of technological development to meet future generation demands, extensive urbanization, globalization and industrialization have resulted in numerous ecological imbalances. This imbalance in the ecosystem has raised numerous global concerns, encompassing water scarcity, climate shift, ozone depletion, environmental contamination, and public health emergencies. The root cause of these global concerns is the deterioration of the environment due to air, water, and land contamination. For instance, contaminated air accounts for 20% of cardiovascular and stroke‐related global mortalities.^[^ ^1^ ^]^ Moreover, World Health Organization (WHO) reports that environmental contamination causes impairment to the human respiratory, nervous and immune systems, which turn humans more vulnerable to high‐risk diseases, especially coronavirus disease (COVID‐19).^[^ ^2^, ^3^, ^4^, ^5^, ^6^ ^]^ Consequently, it has raised the concern of policymakers, environmentalists, the research community, and industrialists to achieve environmental remediation. It can be achieved by monitoring and controlling various contaminants (air, water, radiation, and solid) through specific measures and techniques.^[^ ^6^, ^7^, ^8^ ^]^ Nanotechnology is touted as one of the game‐changing technology to achieve environmental remediation. It consists of architecting various materials and composites with distinctive and superior physicochemical properties at the nanoscale. It is attributed to enhanced surface size effects and quantum confinement effects due to nanoscale confinement of the dimensions of the materials.

**Figure 1:**
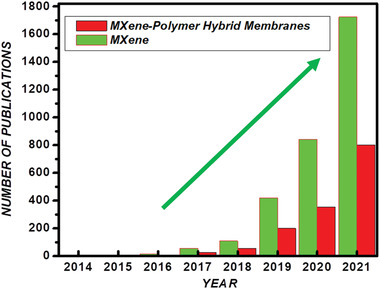
Research and development dedicated to MXenes and MXene–polymer hybrid membranes for environmental remediation applications in the past decade since the discovery of MXenes in 2011. (Analyzed on dimension app with keywords: For MXene, environmental remediation, For MXene–polymer hybrid membrane environmental remediation).

Conventional nanomaterials such as metal and their oxides, carbon nanomaterials, and macromolecules have been utilized for environmental remediation applications such as water purification, desalination, electromagnetic shielding, gas detection and separation, and solid waste reduction.^[^ ^9^, ^10^, ^11^, ^12^ ^]^ However, low remediation efficiency, environmental stability, secondary contamination, and slow and incomplete recovery are the fundamental challenges of these traditional nanomaterials. Over the past two decades, 2D materials, including graphene and its derivatives, metal‐organic frameworks, molybdenum disulfide, phosphorene, silicone, and metal dichalcogenides, have been extensively employed for environmental remediation applications.^[^ ^7^, ^13^, ^14^, ^15^, ^16^, ^17^ ^]^ However, their commercialization has been restricted due to various common processing and storing issues like complex processing, restacking, hydrophobicity, low dispersibility, mechanical flexibility, tedious functionalization, scalability, structural defects, and toxicity. Among them, the last decade has witnessed the rise of metal carbide and nitrides (MXenes) as advanced 2D materials for architecting next‐generation environmental remediation technologies.^[^ ^18^, ^19^, ^20^, ^21^ ^]^ MXene is an emergent class of 2D materials, which are fabricated by selective etching of “A”: 13 or 14 group elements from its precursors, including the MAX phase (with “M” is an early transition material, and “X” can be carbon (C), nitrogen (N), or both), non‐MAX ((MC)*_n_* [Al(A)]*_m_* C_(_ *_m_*_−1)_; *m* is 3,4, and A is silicon (Si) or germanium (Ge)) and “modified‐MAX” phases (like “i‐max phase”; (M^1^_2/3_M^2^_1/3_)_2_AX).^[^ ^22^, ^23^ ^]^ The research and development dedicated to MXenes have exponentially increased in combatting and remediation of air, water, radiation, and solid contamination since its discovery in 2011 (**Figure** **1**).^[^ ^20^ ^]^ The inclusion of MXenes into environmental remediation technologies has witnessed augmentation in its both temporal and spatial features and performances.^[^ ^18^, ^19^, ^20^ ^]^ For instance, high water dispersibility turns MXene into a good candidate for device fabrication and machine processability without leading to any secondary contamination.^[^ ^23^, ^24^ ^]^ It is attributed to the remarkable and tunable physicochemical attributes of MXenes, especially abundant surface functionalities and large specific surface area. Consequently, MXenes have emerged as a popular advanced platform with remarkable environmental remediation performances due to their excellent and tunable conductivities, enlarged specific surface area, rich surface functionalities, high adsorption efficacy, and excellent hydrophobicity, biocompatibility, and significant tribological characteristics. However, pristine MXenes have also been revealed to possess several bottlenecks like easy restacking, low flexibility, affinity towards particular stimuli and poor stability in an oxygen environment, which decreases their lifetime and restricts the commercial development of pristine MXene‐based remediation technologies.^[^ ^25^, ^26^, ^27^, ^28^ ^]^ Despite such remarkable features, the real‐world application of MXenes is limited due to simultaneous requirements of optimization of physicochemical attributes, machine processability, need for flexibility, and robust mechanical endurance.

The drawbacks associated with pristine MXenes have been countered through intercalating its layers, surface engineering, or designing its composites with different foreign materials. Every modification strategy can address specific associated challenges. For instance, oxidation issues can be catered to through surface functionalization, and restacking can be prevented through intercalations.^[^ ^29^, ^30^, ^31^, ^32^ ^]^ Nevertheless, architecting hybrids/nanocomposites are shown to address all these bottlenecks, as the inclusion of secondary material such as macromolecules averts the restacking of MXene layers and introduces mechanical flexibility while retaining substantial mechanical endurance. Besides, the multi‐interactions among the precursors can avert the MXene degradation in an oxygen environment and tune electrical and optical band gaps, optimizing and tuning desired physicochemical attributes.^[^ ^21^, ^33^ ^]^ MXenes have been intercalated or hybridized with other materials such as metals, high‐purity graphene, and its derivative, carbon nanotubes (CNTs), metal oxides, metal sulfides, and macromolecules.^[^ ^34^, ^35^ ^]^ The host–guest chemistries among MXene and other precursors in hybrids/nanocomposites are anticipated to mutually address their drawbacks and introduce unique merits due to synergetic effects and formation of interfacial heterojunctions, leading to high‐performance environmental remediation strategies. For example, Lee et al. demonstrated MXene/reduced graphene oxide (rGO) hybrid to monitor various air pollutants, including ammonia (NH_3_), hydrogen sulfide (H_2_S), sulfur dioxide (SO_2_), ethanol (C_2_H_5_OH), and xylene.^[^ ^36^ ^]^ Though the monitoring efficacy of fabricated hybrid towards NH_3_ detection was manifold superior to that of pristine MXene or pristine rGO, the purification, processing, and functionalization were tedious. Similarly, Hermawan et al. demonstrated copper oxide (CuO)/ Ti_3_C_2_T*_x_* hybrid to monitor low trace toluene optimized at 250 °C, which increases its cost and energy requirement limiting its practical application.^[^ ^37^ ^]^ It points out several bottlenecks associated with inorganic–MXene hybrid‐based environmental remediation strategies, including the requirement of elevated temperature for functioning, tedious processing, toxicity, secondary contamination, and low flexibility.

On the contrary, macromolecules, especially in the form of membranes, have been extensively utilized for devising air, water, radiation, and solid‐waste remediation technologies because of their abundance, flexibility, ease of processing, and room temperature operation.^[^ ^11^, ^22^, ^39^, ^40^, ^41^ ^]^ However, their commercialization is hindered due to a greater affinity towards specific environmental stimuli (like VOCs and humidity), creating cross‐sensitivity and low stability issues, decreasing their operational lifetime. It suggests that encompassing macromolecules into MXenes in the form of hybrids/nanocomposites can cater to the processing and stability issues related to both precursors.^[^ ^29^, ^31^, ^34^ ^]^

Moreover, numerous density functional theory (DFT) and molecular dynamics based studies have shown that MXenes, due to abundant surface functionalities, are prone to link with polymers to form hybrids and nanocomposites.^[^ ^26^, ^42^, ^43^ ^]^ Surface functionalities give rise to multi‐interactions, including covalent, hydrogen bonding, and electrostatic among polymer and MXene precursors resulting in stable hybrids.^[^ ^44^ ^]^ These interactions depend purely on the natures of macromolecules and the surface terminals on MXene. There are reports on the association of several small macromolecules with MXene via covalent bonding because of the strong affinity of MXene towards electron donor groups. Electron‐donating group possessing macromolecules replaces the “T” group of MXene, resulting in forming a hybrid/composite system.^[^ ^45^ ^]^ For example, Tremiliosi et al. showed the presence of covalent bonding in a hybrid of silane‐modified polymers like poly(2‐(dimethylamino)ethyl methacrylate) (PDMAEMA) with MXene.^[^ ^46^ ^]^

Besides, the electrostatic interactions among the negatively charged MXene surface with macromolecule precursors play a considerable role in fabricating MXene–polymer hybrids.^[^ ^47^, ^48^ ^]^ These electrostatic interactions depend upon the nature of the precursor macromolecule. For instance, aniline monomer electrostatically adsorbs over the MXene's surface and between the MXene layers.^[^ ^49^ ^]^

Depending upon various multi‐interactions, there are several reports on architecting MXene–polymer hybrids with various conducting (polypyrrole (PPy), polyvinylpyrrolidone (PVP), polyacrylonitrile (PAN), poly(3,4‐ethylene dioxythiophene) polystyrene sulfonate (PEDOT: PSS)) and hydrophilic (polyimide (PI), polyethylene (PE), polyvinyl alcohol (PVA), polystyrene (PS), silicones, polymers.^[^ ^33^, ^43^, ^49^, ^50^ ^]^ Due to molecular and supramolecular dynamics, attention has been devoted to augmenting and tuning the physicochemical attributes developing from organic–inorganic interfaces in hybrids. Moreover, the synergistic effects due to the inclusion of two different kinds of precursors and hetero‐interfacial effects together contribute to the enhanced performance of MXene–polymer hybrids for numerous applications, especially those devoted to environmental remediation.^[^ ^51^, ^52^ ^]^ Several experimental results have demonstrated that incorporating macromolecules into MXenes forms hybrid/nanocomposites with optimum physicochemical characteristics.^[^ ^43^, ^50^ ^]^ These diversified and enhanced physicochemical properties turn MXene–polymer hybrids into unique materials with high remediation efficacies compared to their precursors and other hybrids. Furthermore, combining polymers in hybrid causes MXene layer exfoliation, increasing interlayer separation and inhibiting MXene stacking.^[^ ^43^, ^53^ ^]^

Additionally, it forms a hierarchal lamellar structured MXene–polymer hybrid and hinders its oxidation in ambient conditions. Moreover, it enhances hybrid systems’ effective surface area and porosity, increasing their interaction with environmental stimuli. Accordingly, MXene–polymer hybrid‐based strategies are promising in diversified environmental remediation sectors, especially in adsorption‐based technologies.

Majorly environmental remediation techniques are devised through designing adsorptive membranes.^[^ ^52^, ^54^ ^]^ Already, polymeric membranes are considered the most breakthrough in membrane‐based remediation technologies due to their low cost, flexibility, abundance, and scalability. However, the swelling in the presence of organic solvents and degradation at elevated temperatures have restricted their commercial viability. MXene–polymer membranes, on the other hand, are the most common type of MXene–polymer hybrids used for reverse and forward osmosis, nano‐, ultra‐, and microfiltration, salination, gas separation, distillation, ion selectivity, catalysis, radiation adsorption, and gas detection. Depending upon the targeted application, these membranes act as selective barriers to restrict, adsorb, or separate the specific ion, gas, radiation, or chemical species. Furthermore, it is due to the tunable physicochemical properties of MXene–polymer hybrid membranes and intact mechanical endurance with mechanical flexibility. These features can be tuned by optimizing the precursors’ nature, concentration, stoichiometry, and surface during the fabrication stage. Though the performances of MXene–polymer membranes are enhanced and superior to pristine precursors, their research and development are still in infancy (Figure 1). Nevertheless, due to the abundance of polymers and the possibility of the varying stoichiometry of MXenes, there is a massive potential in MXene–polymer membranes to meet the industrial requirements for environmental remediation technologies. Hence, it is vital to comprehensively summarize the remarkable remediation features and efficiency of MXene–polymer membranes in the form of a descriptive review.

With this motivation, this review is intended to detail the skeleton of MXene–polymer membranes concentrating on the most fundamental and cutting‐edge progress, including structural control, physicochemical optimization, and application in environmental remediation techniques. This review emphasizes engineering MXene–polymer membranes through various fabrication techniques, their remarkable physicochemical attributes, insight into the trade‐off between precursor concentration and stoichiometry to achieve the desired optimization, and machine processing. Further, the utilization of MXene–polymer membrane to devise environmental remediation techniques has been thoroughly discussed to direct future research by providing this fundamental framework in the form of a review. Moreover, the factors constraining their industrial applications and possible alternate solutions have been presented. Finally, the prospects with cutting‐edge applications with the integration of all advanced technologies like internet‐of‐things (IOTs), artificial intelligence (AI), machine learning (ML), cloud computing, and 5G communications to develop intelligent membranes have been discussed. The synergistic effect between MXenes and polymers opens plentiful opportunities for fundamental exploration and environmental remediation technological applications.

## 2 Engineering MXene–Polymer Hybrid Nanocomposites‐Based Membranes

MXene–polymer hybrid membranes are fabricated using MXenes–polymers as precursors employed by different synthesis strategies. Prior to discussing the MXene–polymer hybrid membrane fabrication, it is essential to understand the fundamentals of MXene fabrication. It is because presynthesized MXenes are used during architecting MXene–polymer membranes.

### 2.1 Fabrication Strategies for MXenes: First Stage for Membrane Engineering

MXenes are fabricated using different synthesis strategies from their respective MAX, non‐MAX, and i‐MAX phase precursors. The fabrication routes of MXenes can be categorized into top‐down and bottom‐up approaches depending upon the fundamental mechanism, as discussed in the subsequent section.

#### Top‐Down Approaches to Engineer Mxenes

**Figure 2:**
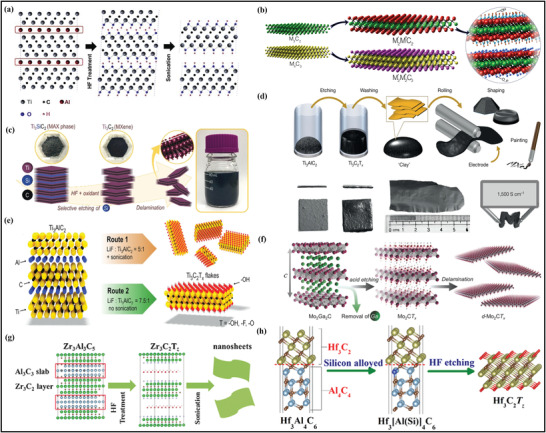
a) Schematic representation of the fabrication of Ti_3_AlC_2_ MAX phase through exfoliation scheme. Reproduced with permission.^[^ ^56^ ^]^ Copyright 2011, Wiley‐VCH. b) Schematic illustration of the new synthesized structures of MXenes. Reproduced with permission.^[^ ^60^ ^]^ Copyright 2015, American Chemical Society. c) Preparation scheme of Ti_3_C_2_ MXene from Ti_3_SiC_2_ MAX phase. Reproduced with permission.^[^ ^67^ ^]^ Copyright 2018, Wiley‐VCH. d) Schematic preparation of the MXene clay process and electrode fabrication. Reproduced with permission.^[^ ^71^ ^]^ Copyright 2014, Springer Nature. e) Synthesis approach of the Ti_3_C_2_ MXene flakes from Ti_3_AlC_2_ MAX phase. Reproduced with permission.^[^ ^73^ ^]^ Copyright 2016, Wiley‐VCH. f) Schematic illustration representing the fabrication and delamination of Mo_2_CT*_x_*.^[^ ^74^ ^]^ g) Fabrication process of the Zr_3_C_2_ nanosheets from non‐MAX phase. Reproduced with permission.^[^ ^77^ ^]^ Copyright 2016, Wiley‐VCH. h) Schematic illustration of 2D hafnium carbide MXene synthesis. Reproduced with permission.^[^ ^78^ ^]^ Copyright 2017, American Chemical Society.

The fabrication of MXene through a top‐down approach depends upon the exfoliation of the solid layers, which are further parted into both mechanical and chemical exfoliation. It is based on the selective etching of MXenes from the MAX phases, non‐MAX phases, and other precursors (mixed MAX phases). Mechanical exfoliation of MXene through this approach is usually not an appropriate method to separate the Al layer from M*_n_*_+1_AX*_n_* due to the excellent metallic bonding between the “A” and “M” elements. The formation of metallic bonding among A and M elements is weak compared to that of the “M” and “X” bond, and the MXenes can be fabricated through the selective etching of element “A” with the utilization of heating and fluoride‐based etchant, especially hydrofluoric acid (HF).^[^ ^55^, ^56^, ^57^, ^58^ ^]^ **Figure** **2**a represents the fabrication of the first Ti_3_C_2_ MXene prepared via soaking the powder of Ti_3_AlC_2_ with 50wt% of HF solution for 2 h under an ambient temperature.^[^ ^55^ ^]^ After the selective etching, the final material was washed and centrifuged several times to get the desired fine powder. The overall reaction mechanism of eliminating the A layer from the MAX precursor is described in Equations ((1), (2), (3)).

(1) $${\mathrm{Ti}}_{3}{\mathrm{AlC}}_{2}+3\mathrm{HF}={\mathrm{AlF}}_{3}+\frac{3}{2}H_{2}+{\mathrm{Ti}}_{3}C_{2}$$

(2) $${\mathrm{Ti}}_{3}C_{2}+2H_{2}O={\mathrm{Ti}}_{3}C_{2}{\left(\mathrm{OH}\right)}_{2}+H_{2}$$

(3) $${\mathrm{Ti}}_{3}C_{2}+2\mathrm{HF}={\mathrm{Ti}}_{3}C_{2}F_{2}+H_{2}$$

The overall course of reactions is summarized in Equations ((1), (2), (3)). The third step of the scheme involves etching with HF, which is responsible for producing the hydroxyl (—OH) and fluorine (—F) surface groups on MXene during the reaction. Following this scheme, Anasori et al.^[^ ^60^ ^]^ prepared the sandwiched layer TiC_2_ along with the Mo‐layers and double M‐contain MXene such as Mo_2_TiC_2_ and Mo_2_Ti_2_C_3_ as displayed in (Figure 2b) to explore the 2D MXene materials. Generally, the formation of surface terminals of MXenes (M*_n_*_+1_X*_n_* T*_x_* where T*_x_* indicates the OH, —O, —F, and —H termination) is dependent on the conditions of the HF selective etching step. Therefore, it is essential to observe that the etching environment of MAX phases depends on the size of the synthesized particles,^[^ ^61^ ^]^ their structural morphology, and the atomic bonding.

Similarly, the concentration performed through HF etchant enhances the atomic numbers in the M element, which further yields the most robust bonding between M‐Al elements.^[^ ^62^ ^]^ Furthermore, as compared to the response time (24 h) of the Ti_2_AlC MAX phase,^[^ ^63^ ^]^ the etching response time (90 h) of the Nb_2_AlC MAX phase^[^ ^64^ ^]^ is threefold greater in the presence of 50% HF concentration at an ambient temperature. It is worth mentioning that for the Nb_2_AlC MAX phase, the temperature must be decreased to 40 h when the etchant temperature reaches 55 °C during the HF concentration.^[^ ^65^ ^]^ Usually, having a large number of “*n*” in MXene needs a higher concentration of HF, that further requires a longer etching time to achieve the whole conversion. For instance, the processing time of the Mo_2_Ti_2_AlC_3_ MAX phases is two‐fold longer (96 h) compared to that of the Mo_2_TiAlC_2_ MAX phase (48 h), even when performed with the same etching conditions in a room environment.^[^ ^60^, ^66^ ^]^

To date, the HF etching route has been extensively utilized to fabricate MXene despite HF being the most hazardous etchant.^[^ ^65^ ^]^ In certain circumstances, such as the fabrication of Ti_3_SiC_3_, the bonding between Ti and Si is stronger than between Ti and Al. It is due to the failure of HF acid to eliminate the layer of Si during the etching step. Alhabeb and the group further illustrate this scheme through the selective etching of the Si layer by utilizing an oxidant agent during the synthesis (Figure 2c).^[^ ^67^ ^]^ The fundamental purpose of adding an oxidation agent is to part the bonds between the Si layer and other elements.

In contrast to HF acid concentration, another in‐situ formation of HF has been widely applied to remove the A element using the same reaction mechanism with less hazardous chemicals.^[^ ^68^, ^69^, ^70^ ^]^ For instance, Ghidiu and Lukatskaya^[^ ^71^ ^]^ reported the rapid and efficient approach to producing a high yield of MXene by etching the Ti_3_AlC_2_ phase treated with lithium fluoride (LiF) and hydrochloric acid (HCl), respectively (Figure 2d). On the contrary, Wang et al. utilized the hydrothermal and soaking approach to fabricate the Ti_3_C_2_–MXene from Ti_3_AlC_2_ MAX phase powder in ammonium fluoride (NH_4_F) solution.^[^ ^72^ ^]^ The reaction mechanism for MXene using NH_4_F and HCl/LiF etchant can be illustrated by Equations (4 and 5).

The overall course of reactions is summarized in Equations ((1), (2), (3)). The third step of the scheme involves etching with HF, which is responsible for producing the hydroxyl (—OH) and fluorine (—F) surface groups on MXene during the reaction. Following this scheme, Anasori et al.^[^ ^60^ ^]^ prepared the sandwiched layer TiC_2_ along with the Mo‐layers and double M‐contain MXene such as Mo_2_TiC_2_ and Mo_2_Ti_2_C_3_ as displayed in (Figure 2b) to explore the 2D MXene materials. Generally, the formation of surface terminals of MXenes (M*_n_*_+1_X*_n_* T*_x_* where T*_x_* indicates the OH, —O, —F and —H termination) is dependent on the conditions of the HF selective etching step. Therefore, it is essential to observe that the etching environment of MAX phases depends on the size of the synthesized particles,^[^ ^61^ ^]^ their structural morphology, and the atomic bonding.

Similarly, the concentration performed through HF etchant enhances the atomic numbers in the M element, which further yields the most robust bonding between M‐Al elements.^[^ ^62^ ^]^ Furthermore, as compared to the response time (24 h) of the Ti_2_AlC MAX phase,^[^ ^63^ ^]^ the etching response time (90 h) of the Nb_2_AlC MAX phase^[^ ^64^ ^]^ is threefold greater in the presence of 50% HF concentration at an ambient temperature. It is worth mentioning that for the Nb_2_AlC MAX phase, the temperature must be decreased to 40 h when the etchant temperature reaches 55 °C during the HF concentration.^[^ ^65^ ^]^ Usually, having a large number of “*n*” in MXene needs a higher concentration of HF, that further requires a longer etching time to achieve the whole conversion. For instance, the processing time of the Mo_2_Ti_2_AlC_3_ MAX phases is twofold longer (96 h) compared to that of the Mo_2_TiAlC_2_ MAX phase (48 h), even when performed with the same etching conditions in a room environment.^[^ ^60^, ^66^ ^]^

To date, the HF etching route has been extensively utilized to fabricate MXene despite HF being the most hazardous etchant.^[^ ^65^ ^]^ In certain circumstances, such as the fabrication of Ti_3_SiC_3_, the bonding between Ti and Si is stronger than between Ti and Al. It is due to the failure of HF acid to eliminate the layer of Si during the etching step. Alhabeb and the group further illustrate this scheme through the selective etching of the Si layer by utilizing an oxidant agent during the synthesis (Figure 2c).^[^ ^67^ ^]^ The fundamental purpose of adding an oxidation agent is to part the bonds between the Si layer and other elements.

In contrast to HF acid concentration, another in‐situ formation of HF has been widely applied to remove the A element using the same reaction mechanism with less hazardous chemicals.^[^ ^67^, ^68^, ^69^ ^]^ For instance, Ghidiu and Lukatskaya^[^ ^70^ ^]^ reported the rapid and efficient approach to producing a high yield of MXene by etching the Ti_3_AlC_2_ phase treated with lithium fluoride (LiF) and hydrochloric acid (HCl), respectively (Figure 2d). On the contrary, Wang et al. utilized the hydrothermal and soaking approach to fabricate the Ti_3_C_2_–MXene from Ti_3_AlC_2_ MAX phase powder in ammonium fluoride (NH_4_F) solution.^[^ ^71^ ^]^ The reaction mechanism for MXene using NH_4_F and HCl/LiF etchant can be illustrated by Equation (4 and 5)

(4) LiF+HCl=HF+LiCl

(5) $${\mathrm{NH}}_{4}F+H_{2}O={\mathrm{NH}}_{3}.H_{2}O+\mathrm{HF}$$

Here, the in situ formation of the HF etchant agent also formed the precipitates in the reaction mechanism of (a–c) to get similar outcomes. Moreover, Lipatov et al. continue this mechanism to prepare the monolayer MXene by varying HF concentration. MXene flakes are obtained with insignificant structural and surface nanoscale defects via utilizing the LiF/HCl approach (Figure 2e).^[^ ^73^ ^]^ It is observed that using a 5:1 concentration of LiF–Ti_3_AlC_2_ gives a lesser quantity of MXene flakes while increasing the concentration to 7.5:1 along with the 5 min of shaking increases the MXene flakes quantity significantly. The ammonium hexafluoroaluminate ((NH_4_)_3_AlF_6_) salt was formed when NH_4_F was treated with aluminum fluoride (AlF_3_), and the reaction mechanism is illustrated in Equation (6).

(6) $$3{\mathrm{NH}}_{4}F+{\mathrm{AlF}}_{3}={\left({\mathrm{NH}}_{4}\right)}_{3}{\mathrm{AlF}}_{6}$$

This reaction takes place due to the occurrence of NH_4_^+^ and Li^+^ cations. Therefore, the MXene layers can be intercalated using these generated ions, which enlarges the spacing amidst the MXene layers. Besides preparing various MXenes by combining and mixing different fluoride salts like CsF, KF, LiF, and NaF with H_2_SO_4_ or HCl acids, this performs the potential role in the upcoming exploration of the synthetic methodologies related to MXenes.^[^ ^59^, ^70^, ^73^, ^74^ ^]^

Like MAX phases, MXenes can also be fabricated through non‐MAX. Meshkian et al. were the first to fabricate the MXene from the non‐MAX phase. They fabricated the Mo‐based (Mo_2_C) MXene through the selective etching of the gallium (Ga) layer from the Mo_2_Ga_2_C non‐MAX phase precursor under the presence of 50 wt% HF concentration.^[^ ^76^ ^]^ Later, Halim et al. proposed the fabrication of Mo_2_C flakes extracted from the Mo_2_Ga_2_C precursor via consuming the LiF/HCl and HF‐based reacting agent solutions as mentioned in (Figure 2f).^[^ ^74^ ^]^ Finally, utilizing the non‐MAX phase precursors, Zhou et al. reported the synthesis of Zr_3_C_2_ achieved from the etching of Zr_3_Al_3_C_5_ and the Al_3_C_3_ layers from the non‐MAX phase (Figure 2g).^[^ ^77^ ^]^ The reaction mechanism can be summarized in Equation (7) as follows:

(7) $${\mathrm{Zr}}_{3}{\mathrm{Al}}_{3}C_{5}+\mathrm{HF}\to {\mathrm{AlF}}_{3}+{\mathrm{CH}}_{4}+{\mathrm{Zr}}_{3}C_{2}$$

The formation of Zr_3_C_2_ in Equation (7) will continue to react further with the HF and H_2_O similar to Equations (2 and 3) to eliminate the termination group from the surface of Zr_3_C_2_. Moreover, in another work, this group yields the fabrication of Hf_3_C_2_ through the selective etching of the aluminum‐carbon (Al‐C) layer from the Hf_3_[Al(Si)]_4_C_6_ compound (Figure 2h).^[^ ^78^ ^]^ It is essential to note that Hf_3_[Al(Si)]_4_C_6_ and Zr_3_Al_3_C_5_, both crystals are linked to the family having the formula of M*_n_* [Al(Si)]_4_C*_n_*_+3_ and M*_n_* Al_3_C*_n_*_+2_ where M and n represent Zr or Hf and the number of layers (1, 2, 3, or 4) respectively.

#### Bottom‐Up Strategies to Architect Mxenes

**Figure 3:**
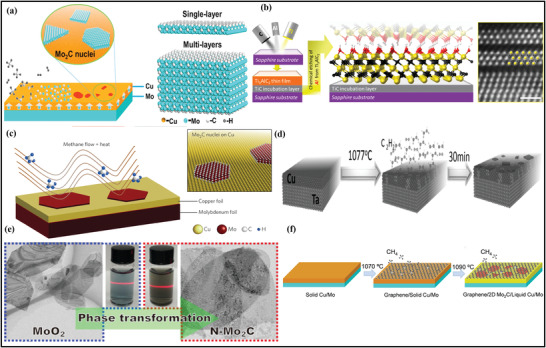
a) Fabrication of MXene on Cu foil. Reproduced with permission.^[^ ^80^ ^]^ Copyright 2019, Springer. b) Epitaxial growth of MXene with the help of magnetron sputtering. Reproduced with permission.^[^ ^81^ ^]^ Copyright 2014, American Chemical Society. c) Fabrication of Mo_2_C MXene nuclei under methane and heat flow through CVD technique. Reproduced with permission.^[^ ^83^ ^]^ Copyright 2014, Springer Nature. d) Schematic representation of TaC MXene thin films. Reproduced with permission.^[^ ^84^ ^]^ Copyright 2017, Wiley‐VCH. e) Phase transformation representation of the preparation of MoO_2_ NS to *β*‐Mo_2_C nanosheets.^[^ ^85^ ^]^ f) Schematic diagram of the CVD growth method of graphene and 2D *α*‐Mo_2_C vertical composite heterostructure. Reproduced with permission.^[^ ^86^ ^]^ Copyright 2017, American Chemical Society.

Bottom‐up fabrication routes are generally employed to fabricate pristine MXene, including chemical vapor deposition (CVD) and atomic layer deposition (ALD) techniques. Xu et al. reported the first fabrication of transition metal carbides and nitrides through the CVD route in which copper (Cu) based metal foil was used as the growth substrate material.^[^ ^79^ ^]^ A schematic illustration of the fabrication scheme is mentioned in (**Figure** **3**a),^[^ ^80^ ^]^ in which Cu/Mo foils are initially heated at a high temperature until the formation of Mo_2_C crystals on the Cu surface. Similarly, Halim et al.^[^ ^81^ ^]^ demonstrated the formation of the Ti_3_AlC_2_ thin‐film MAX phase via depositing Al, Ti, and C elements focused into the sapphire substrate by applying the magnetron sputtering (Figure 3b). The Ti_3_C_2_ films obtained via the CVD route contained 99% light transmittance, having a film thickness of 19 nm, confirming the existence of monolayer MXene‐based thin films. Moreover, like MAX phases, the non‐MAX phase films can also be prepared through the magnetron sputtering tactic, further employed in fabricating epitaxial Mo_2_C growth substrate thin films via easy and effective etching methods.^[^ ^82^ ^]^ It is demonstrated that direct fabrication of ultrathin MXene via the CVD technique can produce large quantities of MXene‐based materials. Like, 2D contained ultrathin MXene (*α*‐Mo_2_C) having the thickness of 3 nm in (Figure 3c) prepared by CVD route under the greater temperature of 1085 °C over the surface of Cu/Mo alloy.^[^ ^83^ ^]^ The thickness and size of the Mo_2_C crystals can be easily manipulated by optimizing the reaction conditions, in which enhancing the lateral size helps to improve the growth time. Similarly, the growth temperature enlarges by increasing the nucleation density.^[^ ^79^, ^83^ ^]^ Wang et al. also reported the 2D‐based Ta‐compounds through the CVD method, where the growth of TaC crystal was obtained via an NH_3_ nitrogen gas source (Figure 3d).^[^ ^84^ ^]^ Like ***α***‐Mo_2_C, Jia et al. reported the bottom‐up synthesis of ultrathin *β*‐Mo2C NS obtained by the fastest and most scalable preparation scheme by utilizing the MoO_2_ NS as a template and Mo as a source.^[^ ^85^ ^]^ Figure 3e shows the schematic SEM images of the phase transformation of MoO_2_ to *β*‐Mo_2_C nanosheets. Xu et al. reported the same methodology to develop the 2D Mo_2_C with graphene‐based vertical heterostructures materials, in which the Mo_2_C is positioned under the layer of graphene (Figure 3f).^[^ ^86^ ^]^ It is important to note that the fabrication of MXenes utilizing the CVD route possesses few defects since no termination group was eliminated during the fabrication process. As a result, synthesizing MXene in this manner provides an intriguing platform for investigating the effect of domain boundaries and characteristics. Therefore, the fabrication of various MXene and multiple functionalities by the bottom‐up approach is essential to be discovered, which further enhances and simplifies the study of their optical and electronic properties.^[^ ^87^, ^88^, ^89^ ^]^

#### Scalable Fabrication of Mxenes for Industrial Prospects

**Figure 4:**
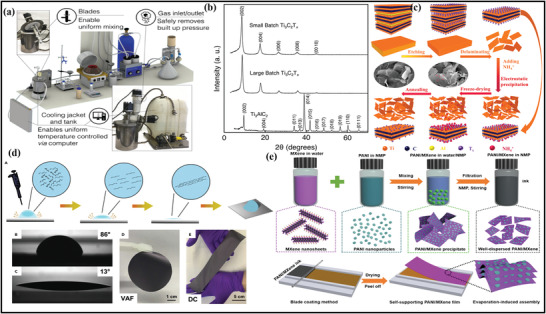
a) Representation of the 3D model of designed MXene reactor for large‐scale manufacture. b) XRD spectra of prepared MXene with MAX phase precursor at small and large‐scale batches. a,b) Reproduced with permission.^[^ ^89^ ^]^ Copyright 2016, American Chemical Society. c) Schematic illustration of preparation of MXene including different MAX phases through freeze‐drying method at large‐scale production. Reproduced with permission.^[^ ^90^ ^]^ Copyright 2020, Wiley‐VCH. d) Schematic illustration of MXene films with its photographic and optical images. Reproduced with permission.^[^ ^92^ ^]^ Copyright 2020, Wiley‐VCH. e) Schematic diagram of the synthesis of PANI nanodots/MXene inks and self‐supporting PANI and MXene‐based flexible films. Reproduced with permission.^[^ ^94^ ^]^ Copyright 2021, AAAS.

Scalable fabrication of MXene and MAX phases is highly essential for multiple applications and commercial viability. With the assistance of a scalable route, large area thin films can be easily produced, which is appropriate for high‐tech and electronic applications. Among all synthesis routes, top‐down strategies are used on a large scale due to ease of availability and high yield, whereas bottom‐up approaches possess limitations of low yield. Shuck et al. were the first to demonstrate the large‐scale manufacturing of MXene by utilizing the custom‐designed chemical reactor shown in (**Figure** **4**a).^[^ ^90^ ^]^ They synthesized the MXene by applying an autochthonic reactor to prepare MXene by comparing it with the MXene case and conventional laboratory‐based chemical exfoliation. The core parts of this reactor include a mixer, gas inlet, gas outlet, cooling jacket, agitator, screw feeder, and thermocouple.

The XRD outcomes in Figure 4b show the same structural characteristics of the small and large‐scale batches of Ti_3_C_2_T*_x_* with dominant peaks.^[^ ^90^ ^]^ Similarly, Zhang et al. obtained the ammonium ion process to monitor and optimize the FL MXene NS considerably.^[^ ^91^ ^]^ The NH^4+^ obtained through solution‐phase flocculation was applied via large‐scale synthesis of MXene and the commercial necessities. Furthermore, this process is also appropriate for the large‐scale progress of different MXenes displayed in Figure 4c.

The scalable fabrication is also required for preparing the MXene hybrids for large‐scale applications.^[^ ^37^ ^]^ However, the research is still in its infancy due to the inefficacy of safer and scalable MXene production routes. Levitt et al. reported using the electrospinning route to fabricate scalable and continuous multifunctional MXene/polyurethane (PU) and nylon nano‐yarns.^[^ ^92^ ^]^ The prepared MXene yarns at scalable production exhibit greater stain performance sensing with the electrochemical properties. However, there is a strong requirement to devise large‐scale manufacturing routes for MXene hybrids, especially green and sustainable approaches.

#### Engineering Mxene Based Films: Scalability in Film Deposition

The fabrication of MXene films (freestanding or substrate‐based) is in high demand for numerous environmental remediation applications. The fabrication of freestanding films formulates the fundamentals of the formation of MXene membranes. They can be fabricated using either top‐down or bottom‐up approaches. For instance, Halim et al. prepared the first transparent MXene thin film (thickness of 19 nm) consisting of magnetron sputtering of MAX phase with a scale size of 1×1 cm^2^.^[^ ^81^ ^]^ The prepared film is utilized in HF and NH_4_HF_2_ solution to obtain the transmittance of 90% and conductivity of about 100 K. Meshkian applied the same approach to fabricate Mo_2_CT*_x_* thin films etched from the Mo_2_Ga_2_C MAX phase.^[^ ^76^ ^]^ During the fabrication of thin films, the various important parameters, including reaction temperature, and plasma monitoring type of substrate, were optimized to obtain continuous MXene films. On the contrary, Lipton reported a drop‐casting method to fabricate the MXene films developed on the hydrophobic plastic substrate.^[^ ^93^ ^]^ The technique includes the deposition of MXene film by pipping out the MXene distribution at the PE film after passing it through the etching process (Figure 4d). The dispersed MXene was pulled strongly over the PE surface and smoothly dried. Furthermore, compared to this route, the fabrication of films can be easily prepared by utilizing the vacuum filtration‐assisted (VAF) strategy, the most straightforward, rapid, and cost‐effective route for architecting freestanding films.

On the other hand, Wan et al. fabricated the MXene films by bridging induced densification to the layered MXene, eliminating voids by forming hydrogen and covalent bonding.^[^ ^94^ ^]^ As a result, the MXene films exhibited excellent electrical conductivity, EMI shielding, and mechanical strength. Moreover, Wang et al. reported the large‐scale fabrication of functional ink of polyaniline (PANI) nanodots with interlayer MXene films, which exhibited excellent volumetric capacitance for the energy storage devices (Figure 4e).^[^ ^95^ ^]^

### 2.2 Engineering MXene–Polymer Hybrid Membranes: Stage Two for Membrane Fabrication

MXene–polymer hybrid‐based membranes have emerged as the most promising strategy for environmental remediation applications. The membrane manufacturing and processing fundamentals included several stages, including MXene fabrication and film formation. Subsequently, the fabrication of MXene–polymer hybrid membrane can be carried out by different routes, which helps enhance the environmental remediation performances of MXene–polymer hybrid materials by optimizing conductivity, stability, mechanical, and optical properties. The subsequent section comprehensively summarizes the different MXene–polymer hybrid‐based membrane fabrication strategies.

#### Architecting MXene–Polymer Hybrid Membrane Using Casting Strategy

The casting route is broadly utilized to fabricate MXene–polymer hybrid membranes with diversified composition, morphology, and structure. In general, minimal amounts of polymer and MXene precursors are mixed in the solvent to obtain the homogenous liquid solution under constant sonication and vigorous stirring. After the sonication stage, the prepared solution is cast into the clean and hygienic substrate to permit the solvent's evaporation at a high rate of temperature under vacuum conditions. After vacuum filtration, the free‐standing synthesized MXene–polymer membrane can be peeled off for further investigation.

**Figure 5:**
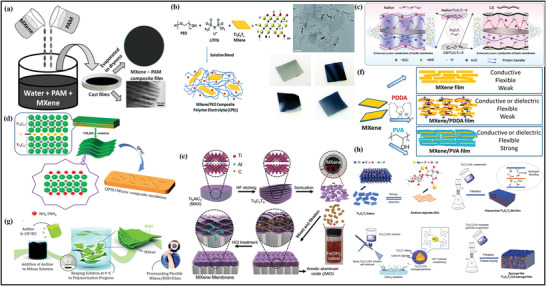
a) Schematic diagram of the casting fabrication of MXene–PAM nanocomposite films. Reproduced with permission.^[^ ^98^ ^]^ Copyright 2017, Elsevier. b) Fabrication and preparation process of MCPEs using few‐layer MXene. Reproduced with permission.^[^ ^96^ ^]^ Copyright 2019, American Chemical Society. c) Schematic illustration of proton transfer through nafion/Ti_3_C_2_T*_x_* and chitosan/Ti_3_C_2_T*_x_* membranes. Reproduced with permission.^[^ ^99^ ^]^ Copyright 2016, Royal Society of Chemistry. d) Synthesis route of the MXene/QPSU composite membranes. Reproduced with permission.^[^ ^101^ ^]^ Copyright 2017, Elsevier. e) Schematics of laminar membrane based MXene–polymer composite by VAF method. Reproduced with permission.^[^ ^104^ ^]^ Copyright 2019, Elsevier. f) Preparation of MXene with PDDA and PVA polymers. Reproduced with permission.^[^ ^105^ ^]^ Copyright 2017, Wiley‐VCH. g) Synthesis approach of MXene/PANI membranes. Reproduced with permission.^[^ ^113^ ^]^ Copyright 2017, Royal Society of Chemistry. h) Preparation of Ti_3_C_2_/SA and Ti_3_C_2_/CA polymer membrane via VAF method. Reproduced with permission.^[^ ^116^ ^]^ Copyright 2016, AAAS.

Due to the hydrophilic behavior of MXenes, and its multi‐interactions with macromolecules, the hydrophilic polymers are specially utilized for the manufacturing and processing of MXene–polymer based membranes using the casting route. These hydrophilic macromolecules largely include polyvinyl alcohol (PVA),^[^ ^96^ ^]^ polyethylene oxide (PEO),^[^ ^97^ ^]^ polyurethane (PU),^[^ ^98^ ^]^ and polyacrylamide (PAM).^[^ ^99^ ^]^ For instance, Saito et al. reported the casting preparation of the MXene–polymer (**Figure** **5**a) in which PAM is merged with MXene and water is used as a solvent to deposit MXene/PAM composite films.^[^ ^99^ ^]^ The obtained Ti_3_C_2_ MXene was further intercalated with dimethyl sulfoxide (DMSO), leading to the MXene–polymer composite dispersion. Moreover, Pan et al. demonstrated the casting route by employing different MXene concentrations incorporated with polymer‐based electrolytic membranes with a mixture of Ti_3_C_2_ and PEO (Figure 5b).^[^ ^97^ ^]^

On the other hand, Liu et al. introduced the same technique for fabricating polymer‐based electrolyte membranes via utilizing the Ti_3_C_2_–MXene merged with chitosan deposited on the glass plate (Figure 5c).^[^ ^100^ ^]^ The fabrication scheme includes separating Al layer via etching from Ti_3_AlC_2_ MAX phase at the initial step, followed by delamination process under the DMSO intercalation. As a result, the Ti_3_C_2_T*_x_* membrane was formed due to casting and ball milling. This work also represents the filler effect of MXene generated on the proton conductivities of Ti_3_C_2_ composite with sulfonated polyether ether ketone (SPEEK) and chitosan polymer membrane. Following this strategy, Zhang et al. reported the fabrication of MXene with sulfonated polyelectrolyte brushes fabricated via utilizing the distillation precipitation polymerization technique.^[^ ^101^ ^]^ The benefit of employing this route is increasing the proton conductivity of the prepared MXene‐SO_3_H membrane. This group also reported the synthesis of MXene with quaterized polysulfone and positively charged sulfone, along with the NH_4_HF_2_ treated with triethylene diamine (Figure 5d).^[^ ^102^ ^]^ Additionally, Ti_3_C_2_‐NH_4_‐QPSU membranes made through the casting process that includes uniformly dispersed MXene have higher ionic conductivity.

Xu et al. also applied the pervaporation route through solvent dehydration to fabricate MXene–chitosan composite membranes.^[^ ^103^ ^]^ The casting method is the simplest and most cost‐effective technique to fabricate films, which primarily depends on deposition and suspension. Until now, this technique has successfully applied most hydrophobic and hydrophilic polymers to prepare MXene/polymer membranes. However, the occurrence of MXene flakes may originate from evaporation synthesis via employing the solvent solution with a higher boiling point.^[^ ^104^ ^]^ Therefore, to prepare the MXene–polymer hybrid, more exertions are required to increase the interaction of MXene among the prepared polymer matrix and MXene flakes, further sorting out to enhance the MXene dispersions inside the polymer matrices. It is important to note that the development of interfacial morphologies between MXene flaxes and polymer matrices was critical in determining how these materials’ chemical and physical properties would work together to build the membrane and effectively manage these structures.

#### Architecting MXene–Polymer Hybrid Membranes Utilizing Vacuum‐Assisted Filtration

Vacuum‐assisted filtration (VAF) method is used to fabricate ultrathin MXene flakes to develop the membrane inside the material. Many successful attempts have been accomplished to prepare the laminar membrane via stacking the MXene and polymer composite illustrated in (Figure 5e).^[^ ^105^ ^]^ Generally, in this method, a specific amount of MXene and polymer are added in a polar solvent under continuous sonification and stirring prior to the filtration. Moreover, Ling et al. reported the PDDA and PVA for the preparation of Ti_3_C_2_–polymer membrane (Figure 5f).^[^ ^106^ ^]^ In addition, various MXene–polymer membrane are fabricated through VAF process like, Ti_3_C_2_‐natural rubber,^[^ ^107^ ^]^ Ti_3_C_2_‐PEDOT: PSS,^[^ ^108^ ^]^ Ti_3_C_2_‐polyflourene derivatives (PFDs),^[^ ^109^ ^]^ Ti_3_C_2_‐aramid nanofiber (ANFs)^[^ ^110^ ^]^ and Ti_3_C_2_‐PEI.^[^ ^111^ ^]^

In addition to the ex situ blending approach used to prepare MXene composites with polymers, an in situ polymerization procedure can also be used to prepare these materials using the VAF method. MXene flakes during the VAF procedure can be readily spread in polar solution through hydrophilic termination, giving the potential premises for the polymerization of various monomers. Normally, MXenes and monomers blended solution is stirred vigorously to finish the polymerization reaction before vacuum‐assisted filtration. Boota et al.^[^ ^112^ ^]^ were the first to report the electrochemically active polymerization of polypyrrole (PPy) as the monomers, while the elimination of group from MXene aided the whole process. The fabricated composite of MXene with PPy reveals the 1000 F cm^−3^ along with the 92% retention due to alignment and intercalation of the PPy chain and the gap between the MXene flakes. Chen et al.^[^ ^113^ ^]^ proposed the same methodology by changing the polymer (EDOT) and proved that the charge transfer of electrons in monomers occurred due to contiguous prospects of Ti_3_C_2_ flakes. Moreover, Vahid Mohammadi and group^[^ ^114^ ^]^ reported the MXene/PANI to observe the thickness of MXene and polymer membrane through this method and showed that varying the volume and concentration of solution thickness can be changed (Figure 5g). Here, PANI was prepared by in situ polymerization, and later freestanding Ti_3_C_2_ merged with PANI having 4–90 µm thickness were produced on a Celgard‐based membrane filter. The fabricated MXene/PANI membrane exhibits a low thickness dependence compared to bare Ti_3_C_2_ films due to intercalation and the hindrance of polymerization chain occurring in the membrane. Therefore, this kind of in situ polymerization eases the dispersion of polymers into the hybrid membrane. The allocation of functionalities over the surface of MXene must be identified for different types of monomers.

Repeatable polymers with outstanding biocompatibility are also highly examined because of their ecofriendly environment, cost‐effectiveness, and mechanical robustness. Due to this, these polymers are the potential contender in preparing MXene and polymer‐containing hybrid membranes.^[^ ^114^ ^]^ Shahzad et al. were the first who introduce the MXene merged with sodium alginate (SA) by VAF procedure owing to oxygen‐based functionalities and the hydrogen bonding formation between the MXene materials.^[^ ^115^ ^]^ Therefore, it displays excellent EMI efficiency by merging both electronic coupling and conductivities of the MXene flakes fabricated by SA. Additionally, using the freeze‐drying and VAF methods, cross‐linked and sponge‐like Ti_3_C_2_ composites with calcium alginate (CA) were created for use in Ti_3_C_2_–SA polymer membranes. Following this, Zhou et al. prepared the Ti_3_C_2_/CA and Ti_3_C_2_/SA polymer membranes to improve the spongy structure and density of the material (Figure 5h).^[^ ^116^ ^]^ Cellulose nanofiber (CNF) was also utilized to fabricate the ultrathin Ti_3_C_2_–CNF composite membrane using the VAF method. Moreover, it is predictable that redeveloped polymers (chitosan^[^ ^117^ ^]^ and polylactic acid (PA))^[^ ^118^ ^]^ can be tested in the manufacturing of MXene and polymer membranes.^[^ ^119^ ^]^ It is shown that the weight percentage composition makes it difficult to fabricate an MXene–polymer membrane using a VAF. As a result, the material processing's final composition differs from the creative design, which further regulates the consistency and relationship between the qualities and composition.

#### Engineering MXene–Polymer Hybrid Membranes through Hot Press Strategies

**Figure 6:**
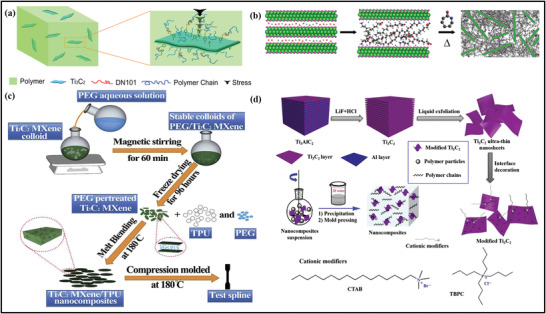
a) Schematic illustration of the mechanism for enhancing the mechanical properties of Ti_3_C_2_ by hot press method. Reproduced with permission.^[^ ^50^ ^]^ Copyright 2016, Elsevier. b) Schematic diagram of the preparation of MXene NCs where lithium intercalated ML Ti_3_C_2_T*_z_* MXene reacts with 12‐aminolauric acid to produce 12‐Ti_3_C_2_T*_z_* to be used for the in situ ring‐opening polymerization of *ε*‐caprolactam. Reproduced with permission.^[^ ^124^ ^]^ Copyright 2019, American Chemical Society. c) Preparation scheme of the TPU/MXene nanocomposites. Reproduced with permission.^[^ ^54^ ^]^ Copyright 2019, IOP Publishing Ltd. d) Schematic representation for the preparation of modified Ti_3_C_2_ ultrathin nanosheets with cationic modifiers, TPU/modified Ti_3_C_2_ nanocomposites, and the chemical structures of cationic modifiers. Reproduced with permission.^[^ ^125^ ^]^ Copyright 2019, Elsevier.

Hotpress method or melt blending is the most preferred and suitable method for fabricating MXene–polymer membrane. It is more developed at an industrial level owing to its ecofriendly environment, flexible formulation, and solvent‐free method. Preparation of MXene flakes can be easily distributed in polymer matrices via blending and controlled temperature, which further acquires the synthesis by hot press route. Most importantly, the obtained composite membrane can be split into the proper dimensions and shapes for evaluation and applications. Similarly, synthesis of MXene‐based hybrid membranes, synthetic strategies, and engineering plastics are intimately entered into our daily routines. Due to its various properties and advantages, ultrahigh molecular weight polyethylene (UHMWPE) is a demanding and excellent performance‐based engineering thermoplastic.^[^ ^121^ ^]^ Zhang et al. applied the hot press route for the preparation of Ti_3_C_2_/UHMWPE along with the various compositions of MXene content as mentioned in (**Figure** **6**a).^[^ ^50^ ^]^ Before applying the bending of UHMWPE for the composition, the MXene surface must be altered by utilizing the isopropyl (dicotylphosphate) titanate to enhance the flakes’ dispersion and compatibility of the material. Therefore, with good mixing speed, MXene flakes were homogeneously distributed inside the membrane, and the resulting Ti_3_C_2_ composite exhibited significant yield strength and hardness. Cao et al. used the linear low density polyethylene (LLDPE) composite with MXene in an open refined rubber apparatus with a temperature up to 125 °C at 20 rpm.^[^ ^122^ ^]^ The prepared MXene nanofillers (NFs) exhibit excellent nonisothermal crystallization impact and thermal kinetics degradation of LLDPE.

In addition, polyurethane and polyethylene have also been used to synthesize MXene–polymer membrane in which colloidal MXene suspensions are essential to be perfectly delaminated to produce the single layer, multilayer, and few‐layer MXene flakes before melt blending with the polymers. Thus, polymer and MXene nanocomposite materials have been restrained to small‐scale fabrication because of the low limit concentration of 20 mg mL^−1^ and predictable polar solvents.^[^ ^123^ ^]^ Carey and co‐workers applied the scalable method for the synthesis of MXene and polymer with multilayered (ML) Ti_3_C_2_T*_x_* materials, as illustrated in (Figure 6b).^[^ ^124^ ^]^ According to the figure, produced MXene flakes are intercalated by the e‐caprolactam monomer and 6‐aminocaproic acid‐based catalyst due to the cation exchange between the 12‐aminolauric acid and Ti_3_C_2_T*_x_* MXene. Thermoplastic polyurethane (TPU) is also extensively utilized due to its excellent deformability and ease of use in industries, medical devices, coatings, and adhesives. It further helps to increase the tensile strength and stiffness of the device when reacting with the MXene materials. Sheng et al.^[^ ^54^ ^]^ used the Ti_3_C_2_/TPU membrane to explore the thermal and mechanical properties of the material prepared through the blending and pressing route as described in (Figure 6c). Ti_3_C_2_ was initially treated with PEG to avoid contamination, then later treated with TPU matrix to increase the device's compatibility. Therefore, Ti_3_C_2_/TPU‐based homogenously spread membranes were found because of the effective PEG intercalation into the layers of MXene. Furthermore, modifying Ti_3_C_2_ via tetrabutyl phosphine chloride (TBPC) and cetyltrimethylammonium bromide (CTAB) improves the hydrophobic interaction inside the polymers. Yu et al.^[^ ^125^ ^]^ prepared the TPU/MXene films with different contents of MXene via co‐coagulation and compression molding process, as shown in (Figure 6d). The as synthesized material yields excellent properties owing to its dispersed catalytic effect of Ti_3_C_2_ flakes, representing the potential physical barriers and applications of these improved cationic Ti_3_C_2_/TPU materials as the strengthening and flame retardant.

In addition, this procedure can easily be utilized without any solvent agent in large‐scale preparation of MXene–polymer membrane. However, this method is not appropriate for polymers with high melting points and less degradation temperature. Similarly, a preprocess of MXene–polymers is required to avoid the aggregation of MXene flakes and to provide excellent dispersion in MXene flakes membranes. Since unexfoliated MXene materials are easier to handle and characterize, it makes sense to construct MXene/polymer membranes from them.

#### Engineering MXene–Polymer Hybrid Using Layer‐by‐Layer Strategy

**Figure 7:**
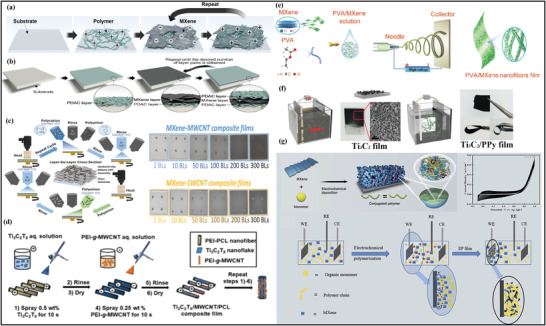
a) Multilayer MXene/polyelectrolyte preparation scheme by LBL method. Reproduced with permission.^[^ ^128^ ^]^ Copyright 2018, Royal Society of Chemistry. b) Preparation scheme of MXene/PDAC membrane. Reproduced with permission.^[^ ^126^ ^]^ Copyright 2014, American Chemical Society. c) Schematic diagram of the spin spray LBL method for synthesizing MXene‐based composite film and bilayer figures of SWCNT and MWCNT with MXenes. Reproduced with permission.^[^ ^129^ ^]^ Copyright 2019, American Chemical Society. d) Schematic illustration of the spray coating LBL method for the preparation of hierarchical MWCNT/Ti_3_C_2_T*_x_* multilayers on the PCL fiber network.^[^ ^127^ ^]^ Copyright 2019, American Chemical Society. e) Fabrication process illustration of MXene–PVA membrane. Reproduced with permission.^[^ ^135^ ^]^ Copyright 2019, Elsevier. f) Schematic diagram of the fabrication of Ti_3_C_2_ and Ti_3_C_2_/PPy film. Reproduced with permission.^[^ ^137^ ^]^ Copyright 2019, American Chemical Society. g) Preparation scheme of the MXene with conjugated polymers along with the cyclic voltammetry and electrochemical polymerization process. Reproduced with permission.^[^ ^134^ ^]^ Copyright 2019, Wiley‐VCH.

The layer‐by‐layer (LBL) route is generally preferred for the functionalized membrane fabrication of MXene, along with meticulous thickness via hydrogen bonding and electrostatic attraction and interaction between the materials.^[^ ^126^ ^]^ Generally, ML films with excellent uniformity can be gained by this method via depositing the opposite species of charges. Due to the negative surface charges, the preparation of MXene and polymer membrane materials was mostly synthesized through LBL‐based dip‐coating approach.^[^ ^127^, ^128^, ^129^ ^]^ Hyosung et al.^[^ ^129^ ^]^ prepared the ML MXene/polyelectrolyte membrane as mentioned in (**Figure** **7**a), synthesized by dip‐coating LBL technique depending on the positively and negatively charged surface of PDAC and MXene flakes. Increasing the number of layers in this route makes the membrane color darker because of the assembly and alteration of MXene/PDAC membrane. Similarly, the thickens of the membrane can be managed on a nanometer (nm) scale, assisting their vital application in ultrafast humidity‐based sensing monitors and devices. Furthermore, like dip‐coating LBL, spin‐spray LBL method shown in (Figure 7b) is also employed to prepare the MXene/PDAC polymer membranes, which exhibit greater conductivity and mechanical properties.^[^ ^127^ ^]^ Weng et al. fabricated the MXene/CNT films by applying the spin‐spray LBL route with PSS and PVA as polymer membrane matrices displayed in (Figure 7c).^[^ ^130^ ^]^ The observed transparency, conductivity, and thickness of the polymer membrane changed by this route, and prepared films revealed excellent conductivity and stability in the water. Zhou et al. utilized this method to synthesize MXene/MWCNT composite. Here, (Figure 7d) shows the preparation scheme of the composite material by spray coating LBL process for the PCL‐based fiber networks and storage applications.^[^ ^128^ ^]^

In addition, the spray coating, spin coating, or dip coating LBL method generally suggests the initial stage towards the excellent performance, portable, wearable, and flexible MXene and polymer‐based electrodes and devices that can be extensively utilized in potential applications like EMI,^[^ ^116^ ^]^ electrocatalysis,^[^ ^131^ ^]^ and sensing^[^ ^132^ ^]^ where ultra and high thin membranes and films along with the supreme exploration of MXene materials to the suitable environment are required.

#### Cutting‐Edge Routes for Architecting MXene–Polymer Membranes

Other fabrication methods of MXene–polymer generally include electrospinning, electrochemical deposition, electrophoretic deposition, and scalpel method. Electrospinning is utilized to prepare nanofibrous or nanofiber films.^[^ ^133^, ^134^, ^135^ ^]^ Jiang et al. prepared the Ti_3_C_2_ MXene with PVA via this approach.^[^ ^136^ ^]^ According to (Figure 7e), the prepared films can be easily twisted, bent, and compressed due to the addition of PVA. Therefore, applying this material as the silk nanofiber films acted as the positive layer and the composite material as the negative layer, a triboelectric nanogenerator was easily produced to use multiple body movements. The same method was adopted to synthesize PVA/polyacrylic acid/Fe_3_O_4_ merged with Ti_3_C_2_ MXene.^[^ ^137^ ^]^

Electrochemical deposition is also used to synthesize MXene–polymer composite materials, mostly to prepare conductive polymer via an in‐situ polymerization route.^[^ ^137^ ^]^ Zhu et al. synthesized the Ti_3_C_2_ with PPy (Figure 7f) prepared via electrophoretic deposition. The mixture, after deposition, intercalated with the pyrrole by in situ polymerization.^[^ ^137^ ^]^ The as‐synthesized material yields excellent stability and capacitance for the all‐solid‐state supercapacitor devices. Qin et al. showed the electrochemical polymerization to prepare the MXene with PPy, and PEDOT membranes can be managed by operating the given amount of current applied on the electrode as mentioned in (Figure 7g).^[^ ^134^ ^]^ Similarly, many microstructures membrane can be managed and gained by the photolithography applied on the conductive substrate. The novel electrochemical deposition gives a unique way to prepare the free‐standing composites of MXene and polymer, along with the electrochemical properties for the storage devices. Compared to all other synthesis strategies, electrochemical deposition requires less human resources, including time, cost, and manpower, and possesses a potential for scalable manufacturing freestanding MXene–polymer hybrids. Moreover, the stoichiometry, chemical composition, nature of reaction parameters, and surface chemistries can be easily controlled and played in electrochemical strategies, which projects it as most potential candidate for MXene–polymer hybrid membranes. **Table** **1** highlights the various MXene–polymer membrane‐based composites with synthesis routes and applications.

**Table 1:** MXene–polymer composite materials with synthesis, application and properties

S. no	MXene/polymer	Synthesis	Application and properties	Refs.
01.	Ti_3_C_2_T*_x_*/EP	Solution casting	Mechanical + EMI shielding	[139]
02.	Ti_3_C_2_T*_x_*/PPy	In situ polymerization	Electrical + supercapacitor	[138]
03.	F‐Ti_3_C_2_T*_x_*/EP	Solution casting	Mechanical + EMI shielding	[139]
04.	rGO‐MXene/EP	Vacuum‐assisted Impregnation	Mechanical + thermal + EMI shielding	[140]
05.	Ti_3_C_2_T*_x_*/TPU	Melt blending	Mechanical + antifriction	[49]
06.	AgNP‐Ti_3_C_2_T*_x_*/Fe_3_O_4_ and PVA	Electrospinning + heat treatment	Wastewater treatment	[137]
07.	Ti_3_C_2_/PLA	Melt blending	Thermal + mechanical	[141]
08.	Ti_3_C_2_T*_x_*/TPU	Melt blending	Mechanical + flame retardant	[54]
09.	Ti_3_C_2_T*_x_* @CS/PU	Dip coating	Pressure sensors	[142]
19.	GO*_x_*/Au/Ti_3_C_2_T*_x_*/naflon	Chemical reduction	Biosensors	[143]
11.	Ti_3_C_2_T*_x_*/PVDF	Vacuum assisted filtration	Antibacterial + wastewater treatment	[144]
12.	Ti_3_C_2_T*_x_*/PDMS	MILD etching	Skin conformal tattoo sensors	[145]
13.	Ti_3_C_2_T*_x_*/PEI modified alginate aerogel	Cross‐linking reaction	Heavy metal ion absorptions	[146]
14.	Ti_3_C_2_/PPy/PET	DIP coating	EMI shielding	[147]
15.	ZnO‐Ti_3_C_2_/paraffin	Hot press	EMI shielding	[148]
16.	Ti_3_C_2_/PVA/MWCNT/PSS	LBL	Electromagnetic absorption	[130]
17.	Ti_3_C_2_T*_x_*/PPy	In situ depositing	Water remediation	[149]
18.	Ti_3_C_2_T*_x_* @Au/polydopamine	Polymerization	Photothermal + catalytic activity	[150]
19.	Ti_3_C_2_T*_x_*/PVDF	MILD etching	Water purification	[151]
20.	Ti_3_C_2_T*_x_*/PA	In situ interfacial polymerization	Water desalination	[152]
21.	e‐PTFE/Ti_3_C_2_T*_x_*	–	Oil spills + waste water	[153]

Researchers are still making countless efforts to introduce novel approaches for preparing MXene–polymer materials. On the other hand, many efforts have successfully improved the MXene and polymer interactions regarding the surface modification of MXene flakes. Furthermore, exploring van der Waal interaction, hydrogen bonding, covalent bonding, and electrostatic interaction, especially complexation interaction, supramolecular interaction, and coordination interaction to synthesize MXene–polymer membranes.^[^ ^126^ ^]^

## 3 Advanced Properties of MXene and MXene–Polymer Hybrids Membranes

### Advancements in Morphological, Structural, and Surface Attributes of MXene–Polymer Hybrid Membranes

**Figure 8:**
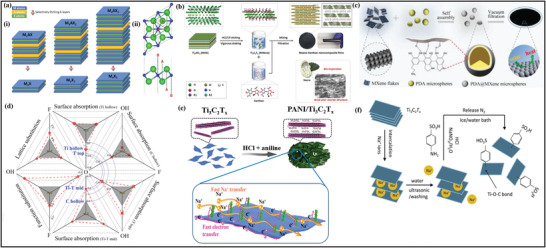
a‐i) Schematic representation of MAX phases to MXene. ii) Upper and lower side views of the M_2_X MXene model. a,b) Reproduced with permission.^[^ ^156^ ^]^ Copyright 2014, American Chemical Society. b) Schematic display of the fabrication process of Ti_3_C_2_T*_x_* MXene/xanthan nanocomposite films. Reproduced with permission.^[^ ^157^ ^]^ Copyright 2020, Wiley‐VCH. c) Schematic illustration of the PDA@MXENE fabrication. Reproduced with permission.^[^ ^158^ ^]^ Copyright 2021, Elsevier. d) Surface absorption of MXene before and after nitrogen incorporation with different energies and functional groups. Reproduced with permission.^[^ ^159^ ^]^ Copyright 2020, Springer Nature. e) Schematic diagram of preparation process of 3D PANI/Ti_3_C_2_T*_x_*. Reproduced with permission.^[^ ^160^ ^]^ Copyright 2020, Wiley‐VCH. f) Schematic illustration of the formation of diazonium ions functionalization. Reproduced with permission.^[^ ^161^ ^]^ Copyright 2020, American Chemical Society.

MXene and MXene–polymer membranes containing morphological, structural, and surface area properties have been broadly developed due to the preparation of MXenes from MAX phase precursors and, later, its composites with polymer materials. Initially, MAX phase precursors are categorized into two kinds of structures. One structure is related to the densely layered‐stacked material structure, and the other is linked to the display hexagonal lattice structure. According to the (M*_n+_*_1_AX*_n_*) MAX phase expression in which M stands for the transition metal (group VI) elements, A indicates the (group III–IV) elements, *n* is the numeric (1,2, 3) number s, while X is the carbon (C) and nitrogen (N).^[^ ^154^, ^155^, ^156^ ^]^ According to **Figure** **8**a, the MXene and MAX phase structure shows the hexagonal symmetry and *P*6_3_/*mmc* space group. Moreover, the bond formation between M–A is broken by the acid reaction, whereas the bond formation between M–X is complete.^[^ ^157^ ^]^ The most important thing is that the A layer is etched from the MAX phase or precursors by applying the strongest acid solution. As a result, the etched layer structures are transformed into the MXene nanosheets.

MXene and polymer hybrid membrane materials are generally fabricated by direct mixing because of the copious functional (—OH, —O, —F) groups present on the MXene surface. The —OH group possesses hydrogen bonds along with the hydrophilic (carboxyl, amino, hydroxyl) functional groups of polymers. The hydrogen bonding in this mechanism is uniformly dispersed and distributed in the MXene and polymer composites.^[^ ^158^ ^]^ Xanthan, a hydrophilic polysaccharide, made a hydrogen bond with MXene (Figure 8b). In this research, the prepared Xanthan and MXene films were 2.0–6.4 times greater than the bare MXene films. Moreover, mechanical strength was improved between MXene and polymer because of hydrogen bonding, which further assists MXene and composite films in constructing various composites with multiple structures. Zhao et al.^[^ ^159^ ^]^ reported the MXene composite with PDA to fabricate the composite microsphere shown in Figure 8c. After filtration, the synthesized material formed microspheres, which further transformed into thin films. These thin films are also processed into the H_2_O molecules after the solar steam mechanism of PDA/MXene films. In addition, the formation of hydrogen bonding among these composite materials occurs due to the physical mixing and strong molecular interaction, which helps MXene and polymers enhance their structure and mechanical properties for various applications like nanogenerators, batteries, and EMI performance.

Surface modification of MXene and polymer is also related to the MXene's hydrophilicity–hydrophobicity surfaces, which is the ability of MXene material to be distributed into the hydrophobic, hydrophilic nature of the polymeric materials. Chemical crosslinked also takes part beside the MXene and polymer surfaces. For example, nitrogen (N) doping is useful for the nitrogen‐based functional (—NO_2_ and —NH_2_) groups on the surface of MXene. Lu et al. reported the MXene with urea to produce the N‐functional group where the formation of Ti—N bond exhibits the interlayer spacing and oxidation state of titanium layer with MXene.^[^ ^160^ ^]^ Figure 8d represents the formation of energy rate of MXene supercell with N‐atoms, attributing to the bond between MXene and nitrogen. Before and after incorporating N, the bond formation indicates the variation of functional groups with lattice substitution where Ti_3_C_2_O_2_, Ti_3_C_2_(OH)_2_ achieve the energy of ‐1.31 and ‐0.09 eV, respectively. Compared with the N‐Ti bond, Wang et al.^[^ ^161^ ^]^ reported the bond formation between MXene with PANI fabricated through an in‐situ polymerization route displayed in Figure 8e, in which the formation of Ti—N bond highlighted takes place amidst the hydroxyl surface of MXene and amino of polyaniline. Moreover, due to the covalent bond formation between Ti—N, the fabricated composite material needed broad interlayer spacing with moderate structural durability to enhance the rapid transfer of ions and electrons, high‐performance energy storage, and less transfer of charge resistance Figure 8f.^[^ ^162^ ^]^ **Table** **2** represents the MXene and polymer composite's structure and surface chemistries.

**Table 2:** MXene/polymer composites with structure and surface chemistry

S.no	MXene/polymer	Structure	Surface chemistry	Refs.
01.	Ti_3_C_2_T*_x_*/PFDTMS	Vertically Janus	Hydrophobicity	[163]
02.	Ti_3_C_2_T*_x_*/Poly(maleic acid)	Channel	Hydrophilicity	[164]
03.	Ti_3_C_2_T*_x_*/PVA	Porous network	Hydrophilicity	[165]
04.	Ti_3_C_2_T*_x_*/PFDTMS	**–**	Hydrophobicity	[166]
05.	Ti_3_C_2_T*_x_*/PDA	Core–shell nanosphere	Hydrophilicity	[159]
06.	Ti_3_C_2_T*_x_*/PDMAEMA	Accordion	–	[167]

### 3.1 Advancements and Tuning in Electrical, Thermal, and Mechanical Attributes of MXene–Polymer Hybrid Membranes

The electrical properties of the MXene–polymer membrane depend on the conductivity of the materials. Generally, polymers behave as an insulator, and the electrical conductivities of the material can be enhanced by adding the MXene flakes to polymers. The intercalation of polymer materials into the layers of MXene will not be enough to assist in the delamination of the ML flakes of MXene. However, it helps accumulate the molecular level bonding and coupling among the polymer and MXene. Ling et al. showed the 0.04–2.2 × 10^4^ S m^−1^ electrical conductivity of Ti_3_C_2_/PVA membrane. Naguib was the first who study the electrical conductivity of MXene via merging with the PAM membrane by utilizing the power law equation.^[^ ^99^ ^]^

(8) $$\sigma =k{\left(m-m_{\mathrm{th}}\right)}^{a}$$

**Figure 9:**
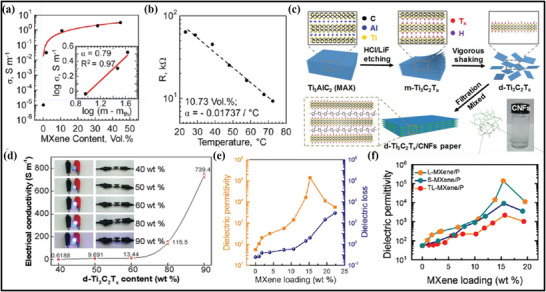
a) Electrical Conductivity of the different MXenes and PAM polymer nanocomposite membrane films as a function of MXene content loading. b) Temperature dependence resistance curve MXene/PAM nanocomposite film. a,b) Reproduced with permission.^[^ ^99^ ^]^ Copyright 2016, Royal Society of Chemistry. c) Representation of the fabrication method of the d‐Ti_3_C_2_T*_x_*/CNFs composite paper. d) electrical conductivity Vs d‐Ti_3_C_2_T*_x_* content for the d‐Ti_3_C_2_T*_x_*/CNFs composite paper sheets with different d‐Ti_3_C_2_T*_x_* contents. c,d) Reproduced with permission.^[^ ^168^ ^]^ Copyright 2018, American Chemical Society.e) Dielectric permittivity Vs dielectric loss of the Ti_3_C_2_/P(VDF‐TrFE‐CFE) membranes. f) Dielectric permittivity of Ti_3_C_2_/P(VDF‐TrFE‐CFE) membranes with large, small, and termination‐rich large MXene flakes versus different MXene content. e,f) Reproduced with permission.^[^ ^170^ ^]^ Copyright 2019, American Chemical Society.

According to Equation (8), *σ* indicates the electrical conductivity of the tested materials, *k* represents any constant, while *m* and *m*_th_ indicate the loading amount of MXene and percolation threshold for enhancing conductivity, and power *a* is the scaling exponent. According to **Figure** **9**a, the electrical conductivity of the composite material is enhanced by increasing the loading content of MXene because of the formation of the percolation network of the MXene flakes. Similarly, electrical conductivity also varies with the environmental temperature in which Ti_3_C_2_/PAM membrane in (Figure 9b) linearly affects the decrease in resistance by increasing the temperature factor. Here, an increase in the electrical conductivity yield the PAM thermal expansion by increasing the temperature of the material. Cao et al.^[^ ^168^ ^]^ prepared the Ti_3_C_2_/CNF composite, as illustrated in Figure 9c,d, where this sample yields the highest electrical conductivity of 739 S m^−1^. Tu et al. also studied the dielectrically increase permittivity of the MXene–polymer based materials by introducing the MXene flakes into the P(VDF‐TrFE‐CFE) matrix.^[^ ^169^ ^]^ Here, according to (Figure 9e), the dielectric permittivity of the materials is enhanced by decreasing the MXene loading content.^[^ ^170^ ^]^ This rapid permittivity change represents the limits and percolation size of the fabricated MXene/polymer hybrid membranes.^[^ ^171^, ^172^ ^]^ Furthermore, the surface and size elimination of MXene material also affects the dielectric permittivity of the MXene/(P(VDF‐TrFE‐CFE)) membrane.^[^ ^170^ ^]^ In this report, MXene flakes exhibit the 4.5 µm dielectric permittivity value, which was ten times greater than the composite hybrid membrane, as shown in Figure 9f. In addition, Mirkhani et al.^[^ ^96^ ^]^ also noted the dielectric constant of MXene with PVA membrane where Ti_3_C_2_ flakes were perfectly aligned into the membranes, and also care‐like structure was formed by VAF methodology to increase the dielectric permittivity and interfacial polarization of the materials. These rapid increments of the dielectric constants and materials’ permittivity and conductivity promote the MXene–polymer application in energy conversion and electromechanical transition.^[^ ^173^, ^174^ ^]^

**Figure 10:**
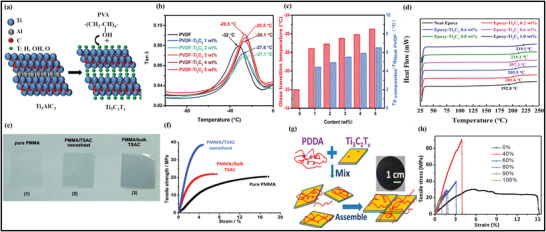
a) Schematic representation of MXene/PVA membrane.^[^ ^177^ ^]^ b) thermal conductivity versus weight content graphical analysis of MXene with PVDF. c) Glass transition temperature (*T*_g_) analysis.b,c) Reproduced with permission.^[^ ^178^ ^]^ Copyright 2017, Royal Society of Chemistry. d) Differential scanning calorimetry (DSC) analysis. Reproduced with permission.^[^ ^180^ ^]^ Copyright 2019, Springer Nature. Standing flexible MXene films with 1 cm of thickness and tensile stress and strain curves for Ti_3_C_2_T*_x_*/PVA films with various Ti_3_C_2_T*_x_* content. Reproduced with permission.^[^ ^106^ ^]^ Copyright 2014, National Academy of Sciences.

The thermal properties of MXene–polymers are closely linked to the application and processing of the materials, together with crystallization, thermal conductivity, and degradation. Thermal conductivity usually defines the charge carrier mobility and the thermal transport property. According to the density functional theory (DFT) calculations, the expected thermal conductivity properties of MXenes are greater than the metals and low‐dimensional semiconducting materials, representing the additive application to increase the thermal conductivity of polymer‐containing composites.^[^ ^172^, ^175^, ^176^ ^]^ Liu and Li^[^ ^177^ ^]^ prepared the Ti_3_C_2_/PVA membrane illustrated in (**Figure** **10**a), where fabricated composite film exhibits the thermal conductivity of 47.6 W m^−1^ K^−1^, and similarly, pristine Ti_3_C_2_ showed 55.8 W m^−1^ K^−1^. Generally, the thermal conductivity of these composite materials is exaggerated by the phase structure distribution, loading, and interfacial thermal resistance. Cao et al.^[^ ^178^ ^]^ investigated the loading content effect of MXene with the composite of PVDF where by varying the less than 0.1 wt% of MXene, thermal conductivity spontaneously enhanced, as shown in Figure 10b. This happens due to the greater Ti_3_C_2_ flakes surface and the hydrogen bonding formation amidst the PVDF and Ti_3_C_2_. In this case, with increasing content of Ti_3_C_2_ interfacial thermal resistance decreases, which is beneficial for the thermal conductivity enhancement of PVDF/Ti_3_C_2_ membranes. Cao et al.^[^ ^122^ ^]^ also prepared the Ti_3_C_2_ with LLDPE membrane where the degradation temperature of this material was 10 °C greater than the clean LLDPE prepared films. In addition, due to thermal degradation of the Ti_3_C_2_ flakes, activation energy and thermal stability of fabricated material increased, which further resulted in decreased frequency. Zou et al. also examined the thermal degradation of MXene with epoxy membrane via thermogravimetric results.^[^ ^179^ ^]^ Moreover, MXene nanofiller also confines polymer chain mobility, impacting the crystallization behavior and glass transition (*T*_g_) of the polymer. Cao et al.^[^ ^178^ ^]^ proposed loading the MXene content on the *T*_g_ temperature of MXene and polymer membrane via studying the dynamic mechanical analysis (DMA), as displayed in Figure 10c. It was observed that the value of *T*_g_ was enhanced for composite membrane by improving the loading amount of Ti_3_C_2_ on it. Similarly, Kang et al.^[^ ^180^ ^]^ also experimented with the same technique by applying the epoxy membrane with Ti_3_C_2_, where *T*_g_ values were noted via differential scanning calorimetry technique, as shown in Figure 10d. The *T*_g_ graph of the composite material is gradually enhanced by adding the content of Ti_3_C_2,_ ascribing the molecular movement restriction and thus increasing the cross‐linking density.

Like thermal properties, mechanical properties are also the most important parameter for polymer‐based membranes and composites due to the gradually improved accumulation of inorganic nanofillers. For example, Zhang et al.^[^ ^181^ ^]^ reported the utilization of Ti_3_Si_0.75_Al_0.25_C_2_ (TSAC) exfoliated NS acted as filler in PMMA membrane Figure 10e–f. The Young's modulus of this composite material was five times pure compared to pristine PMMA film and two times pure of the TSAC/PMMA film with bulk TSAC. This exploration of composite film in which MAX phase is utilized as an inorganic nanofillers gives a new way to apply MXene and composite materials. Similarly, the theoretical prediction of various elastic moduli of MXenes was greater than 500 GPA, indicating the polymeric composite reinforcement. Ling was the first to prepare the polymer and MXene membrane by combining the PVA and PDDA with Ti_3_C_2_.^[^ ^106^ ^]^ Here, Figure 10g represents the fabrication procedure of free‐standing, flexible MXene films, while Figure 10h shows the tensile strength relation in which MXene/PVA composite materials have 40 wt% yields the tensile strength of 91 MPA, while the PVA and Ti_3_C_2_ exhibited the tensile strength of 30 and 22 MPA, respectively.

The mechanical properties of the MXene materials were also enhanced with the better utilization of the nanofiller dispersions via applying less load content of the MXene material. As a result, natural rubber films containing 6.71 vol% Ti_3_C_2_ MXene have 150 times the modulus and 7 times the tensile strength of virgin natural rubber films.^[^ ^107^ ^]^ Moreover, the electrostatic attraction and repulsion amidst the natural rubber and MXene facilitated the MXene flakes dispersions and uniform creation of the interconnect platforms, ascribing the Ti_3_C_2_ and natural rubber membrane strengthening. Sheng et al. reported the Ti_3_C_2_/TPU composites achieved the storage modulus and tensile strength of 39.8% and 47.1%, having the 0.5 wt% loading content of MXene.^[^ ^54^ ^]^ Similarly, Zhang et al. prepared the Ti_3_C_2_/UHMWPE membrane to examine the material's mechanical properties.^[^ ^50^ ^]^ It was reported that this material's breaking and tensile strength are enhanced by increasing the mass fraction of MXene (0.75 wt%). It is mentioned that the enhancement in the mechanical performance of the MXene/polymer membrane is attributed to grain deformation, crack deflection, and grain delamination. Furthermore, the addition in the Ti_3_C_2_/UHMWPE mechanical performance increases its resistance to scratching and plowing, imparting the MXene films a planar worn surface as compared to the UHMWPE films.^[^ ^50^ ^]^ In addition, the MXene–polymer membrane and mechanical properties relationship has been extensively studied at the industrial level. However, several unresolved issues exist regarding the MXene size effect, stress transfer process, MXene mechanism, and MXene flakes distribution and dispersion.

### 3.2 Surface Chemistry, Stability, and Multi‐Interactions in MXene–Polymer Hybrid Membranes

Stability, surface termination, and multi‐interaction are important factors that occur during the MXene fabrication, affect the MXene properties and unlock the various practical applications. Among termination, oxygen is an appropriate element for the electrochemical energy, photocatalysis and hydrogen evolution reaction (HER).^[^ ^182^ ^]^ Surface termination varies during the post‐treatment or storage process.^[^ ^183^, ^184^, ^185^ ^]^ Due to this, Xie et al. observed that the functional group —F could be changed with the hydroxyl (—OH) termination group via storing and rinsing the water, while —OH and —O groups remained stable.^[^ ^184^ ^]^ The —O and —OH are the key termination groups of chemical‐etched MXene, which are stored in solutions with large adsorption capacities. Li et al. predicted the pseudocapacitance of MXene in which Ti_3_C_2_T*_x_* capacitance was three times greater than the bare MXene after terminating the —OH and —F groups via K^+^ intercalation.^[^ ^183^ ^]^ Persson also observed the —F and —OH functional groups that can be transformed into —O termination at a high‐temperature process since oxygen atoms are completely stacked on top of titanium atoms thermodynamically.^[^ ^185^ ^]^ In addition, this —OH and —F elimination reduction is useful to increase the gravimetric capacitance of MXene‐based electrolytic material to hinder the ionic and charge transport capacity.

It is worth mentioning that the MXene flakes oxidation via thermal process could be easily examined via transmission electron microscopy (TEM) and scanning electron microscopy (SEM), in which the prepared materials are further verified by the Raman characterization, X‐ray diffraction (XRD) and X‐ray photoelectron spectroscopy (XPS) examination.^[^ ^186^, ^187^, ^188^ ^]^ Ghassemi et al. reported the MXene flakes oxidations prepared by an in situ environmental TEM where the formation of NPs stacked over the Ti surface during the flash oxidation. On the other hand, a slow heating process converts the Ti layers into nanocrystalline material.^[^ ^188^ ^]^ Moreover, the stability of MXene due to greater temperature conquered through the environmental changes and compositional changes because of the phase diagrams MXene flakes exhibits excellent stability. Furthermore, it is noted that the heating process and temperatures can well balance materials obtained by oxidation. For example, Zhang et al. reported the examination of TiO_2_ in which structural and formation of TiO_2_ are moderately oxidized during the formation of MXene.^[^ ^189^ ^]^ Similarly, Dong et al. reported the hydrothermal approach for preparing potassium titanate and sodium titanate NRs via oxidation and alkalization of the MXene.^[^ ^187^ ^]^

### 3.3 Advanced Attributes of MXene–Polymer Hybrid Membranes

Besides of its all above mentioned outstanding properties of MXene/polymer‐based composite materials, other properties of these materials are generally flame retardancy, antibacterial properties,^[^ ^189^ ^]^ electrode applicability,^[^ ^189^ ^]^ proton conductivity, and antiflame,^[^ ^190^ ^]^ filtration and separation,^[^ ^191^ ^]^ EMI performance,^[^ ^190^ ^]^ and catalytic properties.^[^ ^192^ ^]^ Liu et al. mentioned the MXene composite with PEDOT: PSS to evaluate the EMI shielding performance of the material where Ti_3_C_2_T*_x_*/PEDOT: PSS yields the 1.95 × 10^4^ dB cm^2^ g^−1^ shielding efficiency.^[^ ^190^ ^]^ Yu et al. applied PU with MXene to enhance the flame‐retardant properties of the polymer.^[^ ^124^ ^]^ They added MXene content of 2 wt% along with the tetrabutyl phosphine chloride decreases the heat peak release rate, peak smoke yield, carbon dioxide (CO_2_), and carbon monoxide (CO) by 52%, 57.4%, 51.7%, and 41.6% of TPU, respectively.,^[^ ^99^ ^]^ which exhibits the Grotthuss and vehicle kind proton transfer. Gao et al. reported the fabrication of Ti_3_C_2_T*_X_*/PAN NFs composites to evaluate the removal of PM_2.5_ in the air.^[^ ^191^ ^]^ These countless attempts highlight the extensive performance of the MXene and polymer membrane nanocomposite materials.

## 4 Challenges and Potential Solutions in Architecting MXene–Polymer Hybrid Membranes

Many challenges and hurdles occurred during the fabrication of MXene and its composite materials for potential applications. The initial is to determine the starting and ending quality of the MAX phase materials. Selecting the precursors with excellent quality that are deprived of any extra unreacted phases and substances is crucial.^[^ ^194^ ^]^ Furthermore, MAX phase preparation requires fewer impurities from the mechanical ball milling route.^[^ ^195^ ^]^ These extra impurities can be terminated as sediment during the etching, exfoliation, and delamination. It is noteworthy that if all these materials are soluble in an acidic solution, they can be easily washed and centrifuged during the rinsing period.

Therefore, to overcome this, different situations are employed for MAX precursors during the etching process of MXenes, which helps the material eliminate and solubilize the Al layer from Ti_3_AlC_2_ MAX phase. One of the most difficult challenges was using the hazardous HF etchant, which many researchers are still investigating. The mild method was produced on the optimized condition and nonhazardous etchant. The same process was also applied by in situ formation of HF from the LiF and HCl mixture.^[^ ^196^ ^]^ In addition, Ionic liquids and molten salt are also utilized for the MXene fabrications due to being less hazardous.^[^ ^197^ ^]^

The third issue concerns the delamination method, in which a large number of MXene flakes and grains are not completely delaminated, necessitating a longer delamination time. If not decreased through sonication, the delaminated MXene flakes can also possess larger dimensions. Moreover, for the characterization process of MXene‐based materials, important parameters to monitor are XRD spectra, conductivity, Raman characterization and surface zeta potentials are necessary. In addition, it is seen that different MXene batches exhibit similar Raman and XRD spectra. Due to this, these techniques are particular to bulk nature material and composition, except for those MXene‐based NSs, which may still vary in their physical properties.^[^ ^196^ ^]^ To solve this problem, the first step is to determine the functional group arrangements by analyzing changes in MXene flakes and dimensions, as well as its surface chemistry, using advanced materials characterization techniques.

The fourth challenge is the washing condition to eliminate all the by‐products obtained during Al etching technique. This phase is generally undefined and completed until the pH of the solution rises up to 6 and 7.^[^ ^196^ ^]^ In this work, the adsorbed inorganic ions of Cl^−^ and Li^+^ for the HCl and LiF etching and delamination. Similarly, organic ions of TMA^+^ for the TMAOH delamination and HF etching process may not be easy to wash out. Moreover, multiple washing, centrifugation, and filtration steps are required for this purpose, and enough time is needed to obtain the desired pH value.

Many other challenges are linked to the various MXene kinds and chemistries. Compared to easily etching and delamination of Ti_3_C_2_T*_x_* MXene through LiF and HCl etchant, Nb_2_CT*_x_* and its batches require etching with HF initially and later delamination with TMAOH and TBAOH. HCl and LiF are less active for these kinds of MXene batches. Similarly, V_2_CT*_x_*, Ti_2_NT*_x_*_,_ and Cr_2_CT*_x_* are also difficult to synthesize via etching and delamination into the monolayer flakes because of the greater oxidation tendency and less stability. Due to this, only ML MXene flakes are suitable for fabrication.

It is observed that the MXene material exhibits extraordinary potential during the preparation of MXene/polymer membrane materials, which performs better than just polymer materials. As a result, the production of MXene and polymers remains a question for researchers, who must first test and describe this novel composite before investigating its application. Furthermore, basic issues, like process integration and mass development, must be considered to compete with the necessary industrial and commercial requirements of these materials.

All these discussed issues are linked to the material's challenges, characterization, and industrial and commercialization. Therefore, careful characterization and rapid oxidation are essential immediately after the fabrication. Most of the techniques are examined under room temperature, some in air or some in the presence of partial water, by taking a specific amount of the sample in which the prepared sample may further oxidize under room temperature and while applying the special equipment. These fluctuations may be pursued by varying the peaks and patterns in Raman and XRD spectra while examining the sample.

## 5 MXene and MXene–Polymer Hybrid Membranes for Environmental Remediation Applications

MXenes membranes have several advantages over other materials due to their unique properties, such as nontoxicity, high thermal stability, adjustable energy gap, ideal crystal structure, adsorption ability, excellent electronic properties, hydrophilic nature, and large exposed surface area.^[^ ^198^ ^]^ Their nanocomposites with polymers have also demonstrated potential in various disciplines like energy storage, biomedicine, sensing, electromagnetic interference shielding, catalysis, and biological and environmental protection.^[^ ^199^ ^]^ Furthermore, MXene and its polymer hybrids have shown significant potential in the field of environmental remediation to combat air, water, solid, and radiation contamination. MXene and its polymer hybrids have demonstrated high environmental remediation performance due to their high specific surface area and abundant surface functionalities. The following section emphasizes on environmental remediation application of MXene and its polymer hybrid membranes utilizing various fundamental phenomena.

### 5.1 Advancements in Air Remediation Performance of MXene and Its Polymer Hybrid Membranes

MXene and its polymer hybrid membranes have been reported for air remediation applications, including adsorption, detection and monitoring of air contaminants, and catalysis. Depending upon these fundamental phenomena, the air remediation techniques devised utilizing MXene and its polymer hybrid membranes can be divided into three categories: adsorption, sensing, and catalysis.

#### Adsorption‐Based Air Remediation Techniques Devised Utilizing MXene and Its Polymer Hybrid Membranes

**Figure 11:**
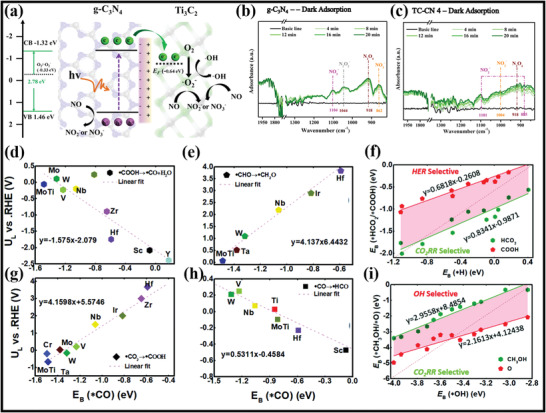
a) Schematic mechanism of hybrid membrane for NO oxidation, IR spectra of O_2_ and NO dark adsorption of b) g‐C_3_N_4_, c) TC‐CN4, The limiting potential (UL) versus RHE relations of hydrocarbon derivatives, the UL versus RHE versus ΔE*CO binding energy were carried out with different catalysts under the same conditions. Reproduced with permission.^[^ ^201^ ^]^ Copyright 2020, Elsevier. The potential determining steps (PDS) for the overall CO_2_RR. Considering that d) *COOH→*CO+*O, e) *CHO→*HCHO, f) which the primary PDS mechanisms in this work for CO_2_RR, Binding energy changes (Δ*E*_B_) for the first protonation step of *CO_2_→*HCO_2_ and *CO_2_→*COOH versus HER. The potential determining steps (PDS) for the overall CO_2_RR. Considering that g) *CO_2_→ *COOH h) *CO→ *CHO. i) which the primary PDS mechanisms in this work for CO_2_RR. Binding energy changes (Δ*E*_B_) for the protonation step of *CH_3_O→*CH_3_OH and *CH_3_O→*CH_4_+*O versus HER. Reproduced with permission.^[^ ^202^ ^]^ Copyright 2020, Royal Society of Chemistry.

Due to rapid population growth and increasing industrialization, gaseous pollutants are extremely harmful to the environment. In general, atmospheric pollutants comprise harmful inorganic gases such as nitrogen oxides, sulfur dioxides, hydrochloric acid, ammonia, and carbon dioxide, as well as volatile organic compounds (VOCs). All these pollutants may cause major health problems for humankind and the environment. These pollutants readily induce infection in the respiratory system, putting several lives at risk.^[^ ^200^ ^]^ Thus, detecting and eliminating them as quickly as possible is essential. Various researchers and research groups have used numerous MXenes and MXene‐derived membranes to remove gaseous pollutants. Ying et al. recently reported the synthesis of a Ti_3_C_2_ MXene‐modified g‐C_3_N_4_ photocatalyst by a straightforward in situ growing procedure (**Figure** **11**a–c).^[^ ^201^ ^]^ The NO removal ratio of the MXene composite structure was much greater (57%) than that of the pure g‐C_3_N_4_ structure. Because of the presence of metallic Ti_3_C_2_, the increased NO removal was ascribed to improved light absorption as well as improved charge transfer, which led to the subsequent adsorption of oxygen molecules on the surface of Ti_3_C_2_ and a reduction in the rate of electron–hole recombination. Li and co‐workers used DFT calculations to forecast the conversion of CO_2_ into methane over MXene nanosheets, which serves as a framework for MXene's importance in renewable energy research.^[^ ^202^ ^]^ As shown in Figure 11d‐i, results revealed that the Sc_2_CO_2_ demonstrated the highest performance of all the materials tested. This performance could be further improved by applying biaxial strains and an external electric field (E‐field). Furthermore, modifying the E‐field makes it possible to achieve simple SO_2_ recovery from Sc_2_CO_2_. It has also been shown that MXene‐based membranes may effectively detect and adsorb various volatile organic chemicals (VOCs), including methanol, ethanol, propanol, acetone, and formaldehyde. In another work, Huang et al. created a membrane of Ti_3_C_2_ that is electrostatically adsorbed on the surface of Bi_2_WO_6_ nameplates, which was then tested for strength.^[^ ^203^ ^]^ The difference in charge density between Ti_3_C_2_ and the volatile organic compounds allows for the ease of charge transfer, hence facilitating the strong adsorption of VOCs and the subsequent oxidation of these molecules. Using fluoride salts (LiF, KF, NH_4_F, and NaF) in HCl, Liu and co‐workers fabricated four types of MXenes and used them as adsorbents for methane gas, among other things.^[^ ^204^ ^]^ Their findings indicated that MXenes created with LiF and NH_4_F could retain methane adsorbed at normal pressure under high‐pressure circumstances, but MXenes prepared with NaF and KF discharged the adsorbed methane at a lower pressure. Furthermore, the MXenes were used to create highly effective nanofiber filters to remove PM 2.5 from the environment.

**Figure 12:**
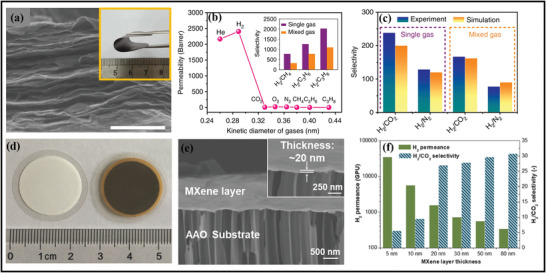
2 mm thick Ti_3_C_2_T*_x_* MXene membranes. a) Cross‐sectional SEM studies of the MXene laminates (scale bar, 1 mm); inset is photo of bent membrane. b) Single gas flux versus a function of the gas kinetic diameter at 25 °C and 1 bar pressure. Inset exhibits the comparative studies of the selectivity of H_2_ gas to the other gases in both single‐ and equimolar mixed gas permeation studies. c) Experimental and simulation studies of selectivities of H_2_/N_2_ and H_2_/CO_2_ and their comparison. Preparation and separation performance of 20 nm thick MXene‐based lamellar membranes. Reproduced with permission.^[^ ^205^ ^]^ Copyright 2018, Springer Nature. d) Digital photos of AAO substrate and MXene nanofilms on a substrate. e) Cross‐sectional SEM studies of MXene nanofilms. f) Gas permeation properties of H_2_ gas and effect of thickness on the separation performance of H_2_/CO_2_. Reproduced with permission.^[^ ^111^ ^]^ Copyright 2018, Wiley‐VCH.

Ding et al. recently developed 2D Ti_3_C_2_T*_x_* laminates with high repeatability for selective gas separation, as shown in **Figure** **12**a–c.^[^ ^205^ ^]^ The permeance of H_2_ gas up to > 2200 Barrer (1 Barrer = 3.348 mol m^−1^ S^−1^ Pa^−1^) and an H_2_/CO_2_ selectivity of > 160 were shown for the first time using these laminates. In order to achieve this remarkable separation capability, the stacked MXene laminates have regular sub‐nanometer channels. These membranes also showed good stability during 700 h of continuous separation of H_2_/CO_2_. These membranes for gas permeation have also been explored in simulation studies, which quantitatively support the findings. H_2_ purification and CO_2_ capture may both benefit from the excellent separation efficiency of these MXene membranes. Shen et al. made MXene nanofilms (20 nm in thickness) and employed them for H_2_/CO_2_ selectivity and separating H_2_ gas, as depicted in Figure 12d–f.^[^ ^111^ ^]^ For H_2_ gas, the membrane has high permeance of 1484 GPU and a selectivity of 27 for H_2_/CO_2_ correspondingly. For CO_2_ gas separation, MXene nanosheets were functionalized with borate and PEI molecules to increase their stacking behavior and interlayer spacing. Using functionalized MXene membranes, CO_2_ gas permeation up to 350 GPU and CO_2_/CH_4_ selectivity of 15.3 were achieved. Furthermore, in a long‐term operating test, MXene membranes, whether pristine or functionalized, were shown to be very stable for up to 100 h. For H_2_/N_2_ separation, Fan et al. found that MXene‐based membranes had a selectivity of 41.^[^ ^206^ ^]^ As a result, their AAO‐supported MXene membrane exhibited no deterioration after 200 h of use. This finding is encouraging for industrially significant H_2_ separation activities. Using MXenes as filler in a poly(ether‐block‐amide) (PEBA) MXene membrane for CO_2_ collection, Gao et al. demonstrated good results.^[^ ^199^ ^]^ Researchers have created high‐performance composite membranes on PAN supports by spin‐coating Ti_3_C_2_T*_x_* nanosheets at a filler loading of 0.15 wt%. The MXene membrane achieved a CO_2_/N_2_ selectivity of 72.5 with CO_2_ permeance of 21.6 GPU due to the preferential affinity of (polar) CO_2_ to (polar) Ti_3_C_2_T*_x_* particles and PEO blocks in PEBA employed. It is also worth noting that the final membrane held up well throughout a 120 h continuous test run. Shamsabadi et al. designed MXene Membrane for CO_2_ collection utilizing two PEBA types (as well as a water‐soluble polyurethane).^[^ ^207^ ^]^ PEO blocks in Pebax 1657 have shown strong selectivity and permeability, surpassing the 2008 Robeson maximum limit for CO_2_/N_2_. However, MXene membranes are stable for six months because the polymer matrix acts as a protective covering, unlike Ti_3_C_2_T*_x_* nanosheets, which deteriorate rapidly in the open air. Although this is not a gas separation application, we believe this is interesting research on removing tiny particulate matter (PM 2.5: atmospheric particles having an effective aerodynamic diameter of less than 2.5 nm) from the air utilizing MXene PAN‐based filters. Effective aerodynamic diameter takes into account both the size of the filter mesh and the attraction of particles to its surfaces when determining how well particulate air filters operate. Accordingly, just 0.005 to 0.080 wt % of MXene was added to PAN by the authors; (by combining MXene and PAN as the electrospinning precursor). Both in terms of capacity (>2 times) and rate (4.2 vs 44 g cm^−2^ h^−1^), the electrospun composite filter produced this higher performance in the removal of PM 2.5. Preliminary results show that the composite filters restricted the development of germs by utilizing Staphylococcus aureus as a model.

#### Advances in Air Contaminant Monitoring and Detection Using MXene and Its Polymer Hybrid Membranes

**Figure 13:**
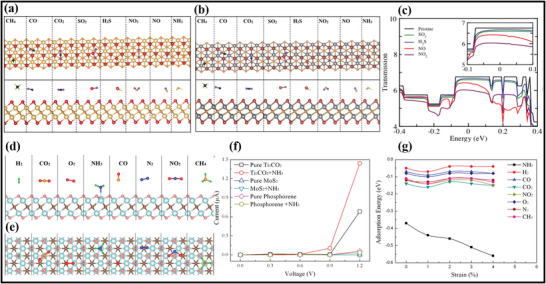
a) The schematic diagram of the sensing mechanism of gases (CH_4_, CO, CO_2_, SO_2_, H_2_S, NO_2_, NO, and NH_3_) on a) Ti_2_NS_2_ b) V_2_NS_2_ MXene sheets. Golden, gray, orange, red, light green, purple, and black spheres represent Ti, V, N, S, H, O, and C atoms, respectively, Zero bias transmission. c) Ti_2_NS_2_ MXene sheets with adsorbed gas molecules (NO, NO_2_, H_2_S, and SO_2_) on the sheet (inset shows the sensitivity around the Fermi level). Reproduced with permission.^[^ ^216^ ^]^ Copyright 2020, Elsevier. A schematic illustration of d) side, and e) top view of the adsorption of NH_3_, H_2_, CH_4_, CO, CO_2_, N_2_, NO_2_ or O_2_ molecule on monolayer Ti_2_CO_2_. f) The calculated *I–V* curve before and after the adsorption of NH_3_ on Ti_2_CO_2_, MoS_2,_ and phosphorene. g) The adsorption energies of gas molecules (including NH_3_, H_2_, CH_4_, CO, CO_2_, N_2_, NO_2,_ or O_2_) on monolayer Ti_2_CO_2_ as a function of applied biaxial strains (from 0% to 4%). Reproduced with permission.^[^ ^217^ ^]^ Copyright 2015, American Chemical Society.

Air contaminant sensing is a fast‐growing technique because of the wide range of applications such as air quality monitoring, pollution management, medicines, diagnostics, and breath monitoring. Gas sensitivity has become essential to contemporary society because of the increasing demand for air quality monitoring, food safety, and public health care.^[^ ^208^ ^]^ It is essential that the gas sensing material has a large surface area for excellent interaction with gaseous molecules, active surface sites for effective and selective absorption of target gas molecules, and a higher capacity to convert the interaction into electrical signals.^[^ ^209^ ^]^ We will compare MXene to typical 2D materials and discuss why MXenes are most suitable for sensor applications before going into detail about several MXene‐based sensors. Besides the benefits of standard 2D materials, MXene features hydrophilicity, flexible layer spacing, abundant surface functional groups, and unique surface chemical characteristics (i.e., facile surface functionalization).^[^ ^210^ ^]^ It also has strong biocompatibility and good conductivity. Low detection limit, fast reaction time, high sensitivity, broad linear range, and great selectivity for a specific gas are all essential features of a high‐performance gas sensor. As a result, many sensor applications have been investigated using MXene. Different sensing devices are now being developed using MXene, including pressure and strain sensors, gas and temperature sensors as well as electrochemical and biosensors.^[^ ^208^ ^]^ Electronic devices may be improved by incorporating MXenes with other materials such as enzymes, metal nanoparticles, conductive polymers, and metal oxides. As well as NO_2_ and H_2_S detection, detecting volatile organic compounds (VOCs) at ppm concentrations is critical for the timely diagnosis of serious disorders. Chemiresistive sensing of a large number of gaseous molecules is controlled by surface adsorption, which produces charge transfer in the interaction of gas molecules with 2D materials.^[^ ^211^ ^]^ For semiconductor gas sensors, the charge carriers are extremely significant. The target gas concentration may be calculated by comparing the number of charge carriers to the density of the charge carriers after exposure to the target gas under particular circumstances.^[^ ^20^ ^]^ When using materials with narrow bandgaps, such as graphene, the adsorption energy of gases is reduced since the charge distribution is not controlled.^[^ ^212^ ^]^ On the other hand, broad bandgap materials like MoS_2_ have high binding energies but are hampered by their poor charge mobility. In contrast to the two 2D materials previously discussed, MXene membranes have a better signal‐to‐noise ratio. It is the adsorption of gas molecules on the surface of MXenes that triggers a change in resistance when they're exposed to air.^[^ ^213^ ^]^ The MXene molecule's absorption energy and the related charge transfer characteristics are essential in determining the gas‐sensing features of MXene.^[^ ^214^ ^]^ When gas molecules contact a solid, they have a certain amount of energy that is absorbed by the substance. Currently, the use of MXene in gas sensors is quite confined. Adsorption‐based gas response or surface functions of MXenes must be differentiated; hence, a greater understanding of sensing processes is required.^[^ ^215^ ^]^ Naqvi et al. reported that M_2_NS_2_ MXenes could act as a gas sensor for toxic air pollutants, enabling them to detect even the tiniest amounts of toxic air pollutants in respired inhalation (**Figure** **13**a–c).^[^ ^216^ ^]^ Also, Yu and co‐workers described monolayer Ti_2_CO_2_ MXene as well suited for extremely efficient NH_3_ sensing because of the different transport properties and the abrupt shift in current–voltage characteristics before and after NH_3_ adsorption.^[^ ^217^ ^]^ The surface of Ti_2_CO_2_ MXene can also adsorb a variety of different gases, including O_2_, CO_2_, N_2_, NH_3_, NO_2_, H_2_, and CH_4_ in order to maximize its potential as a gas detecting material (Figure 13d–g). Liu et al. prepared a biosensor of Ti_3_C_2_ MXene for nitrite detection. It is a decent performer with a linear range of 0.5–11800 µm. According to the findings, the enzyme immobilization technique MXene–Ti_3_C_2_T*_x_* provides a wide range of possible applications for environmental investigation.^[^ ^132^ ^]^

**Figure 14:**
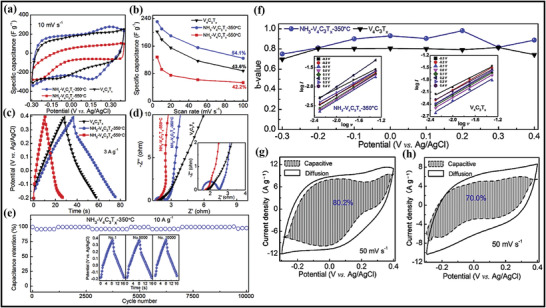
a) CV curves of the V_4_C_3_T*_x_*, NH_3_‐V_4_C_3_T*_x_*‐350 °C and NH3‐V_4_C_3_T*_x_*‐550 °C at 10 mVs^−1^. b) The Cs of V_4_C_3_T*_x_*, NH_3_‐V_4_C_3_T*_x_*‐350 °C and NH_3_‐V_4_C_3_T*_x_*‐550 °C at different scan rates with corresponding capacitance retention. c) GCD curves of the V_4_C_3_T*_x_*, NH_3_‐V_4_C_3_T*_x_*‐350 °C and NH_3_‐V_4_C_3_T*_x_*‐550 °C at 3 A g^−1^. d) Nyquist plots of the V_4_C_3_T*_x_*, NH_3_‐V_4_C_3_T*_x_*‐350 °C and NH_3_‐V_4_C_3_T*_x_*‐550 °C electrodes (the inset: magnified plot in the high‐frequency region). e) Capacitance retention of the NH_3_‐V_4_C_3_T*_x_*‐350 °C electrode (10 000 GCD cycles) at 10 A g^−1^, with the insets showing GCD curves of the NH_3_‐V_4_C_3_T*_x_*‐350 °C after the first, 5000, and 10 000 cycles. f) The plots of *b*‐value versus potential for both the V_4_C_3_T*_x_* and NH_3_‐V_4_C_3_T*_x_*‐350 °C (the inset: log *I* vs log *v* at different potentials for both the V_4_C_3_T*_x_* and NH3‐V_4_C_3_T*_x_*‐350 °C, the slope of each line is the corresponding *b*‐value). The capacitive and diffusion contribution to the overall capacitance of the g) NH_3_‐V_4_C_3_T*_x_*‐350 °C and h) V_4_C_3_T*_x_*. Reproduced with permission.^[^ ^218^ ^]^ Copyright 2019, Elsevier.

Zhao et al. developed a novel V_4_C_3_T*_x_* MXene by employing aluminum and cobalt as catalysts and used V_4_C_3_T*_x_* MXene film to detect the presence of acetone.^[^ ^217^ ^]^ As illustrated in **Figure** **14**a–h, the sensing signal is collected by applying DC voltages across the resistance of the V_4_C_3_T*_x_* film between the two copper electrodes. The sensor is positioned in a vacuum‐sealed container, and the acetone steam is mixed with nitrogen (N_2_) as the carrier gas input via a constant airflow. After HF treatment, V_4_C_3_T*_x_* MXene altered from metal V_4_AlC_3_ to a semiconductor. According to the author, a substantially bigger molecular size difference between water and acetone might explain water vapor's acetone selectivity. V_4_C_3_T*_x_* MXene's acetone‐sensing capabilities are strong, and detection limits are 1 ppm and 25 °C for operating temperature. As one of the few acetone sensors that can match the criteria of low concentration (sub‐ppm), high sensitivity, and high selectivity for mixed gases, V_4_C_3_T*_x_* MXene's good acetone sensing performance opens the door for MXene's use in gas sensors. Likewise, A sensitive fluorescence sensor for the detection of Ag^2+^ and Mn^2+^ ions was developed by Desai and co‐workers. Ag^2+^ and Mn^2+^ have detection limits of 9.7 and 102 nm, respectively.^[^ ^218^ ^]^ Therefore, with excellent recovery, the sensor may detect Ag^2+^ and Mn^2+^ ions in food and water samples. According to the research, MXenes materials have several advantages for environmental analysis, such as pollution and metal ions impregnation.

#### Catalysis‐Based Air Remediation Techniques Devised Utilizing MXene and Its Polymer‐Based Membranes

**Figure 15:**
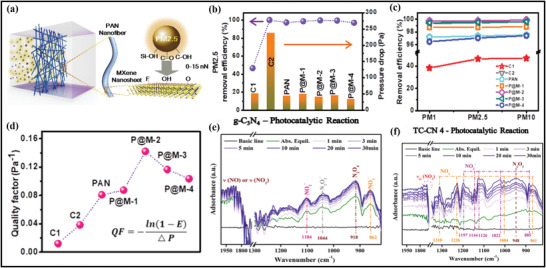
a) Scheme of the enhanced interaction between PM2.5 and MXene nanosheets, b) PM2.5 removal efficiencies *E* and pressure drop Δ*P* of all prepared and the two commercial filters. *E* = (*C*_0_‐*C*_1_)/*C*_0_, where *C*_1_ is the mass concentration of PM in the exhausting gas after the filter and *C*_0_ is the feeding mass concentrations of PM before the filter, c) PM1, PM2.5, and PM10 removal efficiency of all filters. d) Quality factor QF comparison between all prepared and the two commercial filters. Reproduced with permission.^[^ ^192^ ^]^ Copyright 2019, Elsevier. In situ IR spectra of NO and O_2_ photocatalytic reaction e) of g‐C_3_N_4_, f) TC‐CN 4. Reproduced with permission.^[^ ^201^ ^]^ Copyright 2020, Elsevier.

The light energy is initially absorbed during photocatalysis, generating electrons and holes. The produced electrons and holes are then separated and moved to the surface of the photocatalyst.^[^ ^19^ ^]^ Then, reduction and oxidation events occur when the indicated species are consumed. For the homogenous development of catalysts, such as TiO_2_, MXenes operates as strong support for electron–hole separation.^[^ ^220^ ^]^ The large surface functional groups of MXenes have to provide reactants with readily accessible active sites for adsorption. Metal‐organic frameworks may benefit from adding MXene to improve their photocatalytic activity (MOFs).^[^ ^118^ ^]^ The degradation of ciprofloxacin (CIP), tetracycline (TC), RhB, and bisphenol A (BPA) under visible light irradiation was investigated for the possible photodegradation application of graphene layers linked to TiO_2_/g‐C_3_N_4_. It was found that the intensity of the hybrid's emission spectrum was lower than that of a pure species, indicating that the electron‐hole pair had been separated, which is necessary for effective photodegradation.^[^ ^220^ ^]^ By using graphene as a mediator in this system, shortcomings, such as a lack of surface area, light absorption at visible light, and increased photodegradation, were encountered. Gao et al. developed a 2D MXene nanosheet‐modified PAN fiber membrane to purify air.^[^ ^192^ ^]^ The insertion of Ti_3_C_2_ MXene nanosheets into PAN fiber increased the effectiveness of PM2.5 removal to 99.7% with a low‐pressure drop of 42 Pa, while also providing better antibacterial activity **Figure** **15**a–d. MXene nanosheets#x00027; large surface area and easily accessible termination points contribute to the high effectiveness of PM2.5 removal by allowing quick and abundant adsorption of PM2.5 particles via strong contact forces. In another study, Zhou et al. conveyed the fabrication of Ti_3_C_2_/g‐C_3_N_4_ through in situ growth. The NO removal ratio of the MXene heterojunction was 57% greater than that of the bare g‐C_3_N_4_ structure.^[^ ^201^ ^]^ Improvements in light absorption, better charge transfer, oxygen molecule adsorption on the surface of Ti_3_C_2_, and reduced electron–hole recombination were all attributed to the enhanced NO elimination (Figure 15e,f). Similarly, using the ultrasonic reduction process, Liu et al. developed an agglomerated accordion‐like structure of Au nanoparticles anchored with Ti_3_C_2_.^[^ ^221^ ^]^ The NH_3_ yield of 30.6 mg (h mg)^−1^ and the Faraday efficiency of 18.34 percent at 0.2 V of Au/Ti_3_C_2_ were outstanding. High‐valence‐state gold clusters and Ti_3_C_2_ were shown to be the driving force for the weakening of triple N‐N bonds, which was achieved by stabilizing N_2_* species efficiently and disrupting N_2_NH_2_* under an indirect route.

### 5.2 Advancements in Water Remediation Applications of MXene and Its Polymer Hybrid Membranes

Extensive use of water with industrialization, urbanization, and water contamination has raised the serious global concern of water scarcity. It has resulted in inordinate development in water remediation and desalination techniques utilizing nanomaterials. MXene and its polymer hybrid membranes are widely researched and utilized to purify wastewater by filtration and adsorption of water contaminants like heavy metal ions, dyes, and desalination. The following section discusses the advancements and developments in MXene and its polymer hybrids for water remediation techniques based on different fundamental approaches.

#### Advances in Filtration Based Techniques for Water Remediation Techniques

**Figure 16:**
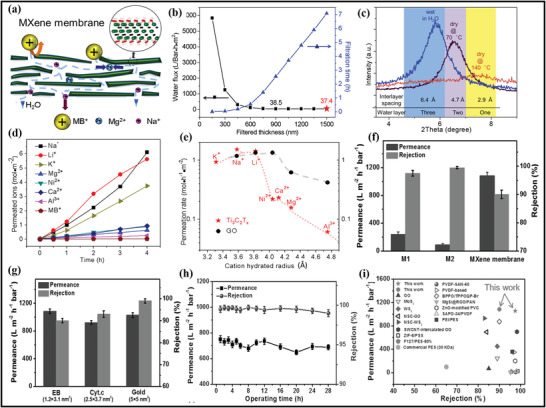
a) Schematic mechanism of MXene membrane. b) Water flux through Ti_3_C_2_T*_x_* membrane with varying thicknesses, and red star marks the thickness of membranes used in ion permeation tests. c) (0002) peaks of XRD patterns of Ti_3_C_2_T*_x_* membranes at different moisture levels and average interlayer spacing calculated from XRD data. d) Number of cations permeated through the Ti_3_C_2_T*_x_* membranes against time for 0.2 mol L^−1^ feed solutions (all solution utilize a Cl^−^ counter anions). e) The permeation rates of cations against their hydration radii through the Ti_3_C_2_T*_x_* and GO membranes. Reproduced with permission.^[^ ^225^ ^]^ Copyright 2015, American Chemical Society. f) Comparison of the performance of the M1, M2, and MXene membranes for the separation of EB molecules at room temperature. g) Separation performance of the MXene membranes for different molecules with different sizes. h) Separation performance versus filtering time for filtration of gold nanoparticles (5 nm) solution using the 1 mm thick MXene membrane. i) Comparison of the separation performance of the MXene membrane and various previously reported membranes, as well as the commercial PES membrane. Reproduced with permission.^[^ ^105^ ^]^ Copyright 2017, Wiley‐VCH.

As a result of the outstanding permeability capabilities of membranes composed of 2D materials, there has been a significant increase in interest in this technology. As a result of their high 2D layer strength, large surface area, hydrophilicity, abundance of terminations, and outstanding flexibility, MXene membranes are an ideal material for wastewater treatment.^[^ ^220^ ^]^ Membranes have several distinct benefits, the most important of which are high selectivity, high flux, and resistance to fouling. Membranes must be thin while being mechanically unaffected in order to enhance the rejection rates of salt and water permeability.^[^ ^221^ ^]^ A variety of 2D materials regulate MXenes#x00027; ability to identify certain impurities, including transition metals like graphene, chalcogenides, and GO, via the interactions between the stacked layers and the molecules being carried. Stacked 2D MXene nanosheets on porous substrates make it easy to make microporous thin films.^[^ ^222^ ^]^ The interlayer spacing between these films varies from 2.9Å in dry conditions and 6.4 Å in wet situations, depending on the wetting conditions.^[^ ^223^ ^]^ Polymer membranes were first utilized as a substrate to support the MXene membrane in its early stages.^[^ ^147^ ^]^ Ren et al. observed an ultrafast water flow and salt selection based on size and charge with the Ti_3_C_2_ membrane supported by PVDF.^[^ ^224^ ^]^ Ti_3_C_2_/PVDF water's flux reduces with increasing thickness. The water flux of Ti_3_C_2_/PVDF membranes is five times greater than that of graphene oxide (GO) membranes of the same thickness because of the presence of water molecules between the Ti_3_C_2_ layers in the wet state. As a result of these findings, the Ti_3_C_2_/PVDF membranes exhibit much lower ion penetration rates for multiple‐charged ions than the Go membrane, and they show higher selectivity for ions, mostly for those which have radii less than 4.5 Å and charges between +2 and +4 (**Figure** **16**a–e). During filtration, small interlayer spacing and negatively charged surface of Ti_3_C_2_/PVDF membrane reject cations with hydrodynamic radii and significant charge. Ti_3_C_2_/polyimide (PI) membrane has been developed by Han et al., with the polymer matrix connected by triethylenetetramine (TETA).^[^ ^225^ ^]^ Using water flux greater than 268 L m^2−^ h^1−^ at 0.1 MPa and ambient temperature, the Ti_3_C_2_/PI membrane rejected gentian violet (100%) while rejecting Congo red (78.5%). Despite Congo red's larger molecular weight, the linear shape of Congo red allows it to pass through the membrane more easily than gentian violet (408 g mol^−1^). Ti_3_C_2_/PI membranes were also shown to be more stable in different solvents, such as dimethylformamide (DMF), acetone (ACN), as well as methanol. With the inclusion of MXene nanofillers, the permselectivity of the Ti_3_C_2_/PI membrane with 1% MXene flakes is significantly improved (67 L m^2−^ h^−1^ at 1.38 MPa) over the Ti_3_C_2_/PI membrane with PEI cross‐linking. High water flow and low salt rejection^[^ ^225^ ^]^ due to the loose structure and enlarged water channel of the Ti_3_C_2_/PI membrane make it suitable for wastewater treatment with reduced osmotic pressure and operating costs. Ding and co‐workers fabricated a Ti_3_C_2_T*_x_*–MXene membrane utilizing the VAF process in order to manage its porosity.^[^ ^104^ ^]^ In the beginning, they intercalated the positively charged iron hydroxide (Fe(OH)_3_) nanoparticles between the negatively charged Ti_3_C_2_T*_x_* sheets, and then VAF was accomplished on the anodic aluminum oxide substrate (AAO). Ti_3_C_2_T*_x_* membranes were then created using an acid solution to dissolve Fe(OH)_3_. For the Ti_3_C_2_T*_x_* membrane, permeability was found to be about 1084 L Bar^−1^ h^−1^ m^−2^, along with a 90% EB dye rejection rate Figure 16f–i. Before dissolving the Fe(OH)_3_ nanoparticles, the membrane had ten and five times greater permeance than the Ti_3_C_2_T*_x_*/Fe(OH)_3_ and pure Ti_3_C_2_T*_x_* membranes. For cytochrome molecules, EB, and gold nanoparticles with a diameter of 5 nm, the membranes thickness of 0.8 µm had a 100% rejection rate. For 28 h, gold nanoparticle filtration was used to study the membrane's stability. It maintained the water permeability and rejection efficiency at a constant level. Despite the high rate of flux, the biggest drawback was the low salt rejection. MXene‐based membranes still exceeded the 2D membranes MoS_2_, WS_2_, and graphene under the same circumstances while still retaining outstanding separation performance. Doping MXene‐based membranes with noble metals may improve their water permeability. Ti_3_C_2_/chitosan membranes for pervaporation dehydration of organic solvents were constructed by Xu et al. using a linked Ti_3_C_2_ membrane, which greatly improved water permeability.^[^ ^102^ ^]^ MXene/polymer membranes with a modest MXene loading of 3 wt% showed outstanding separation factors for ethyl acetate, ethanol, and dimethyl carbonate was around 4898, 1421, and 906 with a water flux of 1.5 kg m^2^ h^−1^, showing considerable potential for pervaporation dehydration. Similarly, a colloidal Ti_3_C_2_T*_x_* membrane with 0.45 m pores was designed by Ren and co‐workers using vacuum‐assisted filtration (VAF) to reject molecules and ions based on their sizes and charges.^[^ ^226^ ^]^ High water flow (37.4 L Bar^−1^ h^−1^ m^−2^) and excellent ion separation dependent on ion charges and hydration radius were shown by the micrometer‐thick Ti_3_C_2_T*_x_* membrane. According to the filtration observations, the micrometer‐thick Ti_3_C_2_T*_x_* membrane may diffuse monovalent cations (Na^+^, K^+^, Li^+^) more easily than multivalent ions (Mg^2+^ and Al^3+^). Additionally, monovalent cations showed a higher penetration rate than ions with larger hydration radii and charges. MXenes have the ability to degrade contaminants when exposed to a certain amount of maintainable sun radiation, a process known as photodegradation. MXene/polymer membranes with higher separation performance may be discovered by systematically controlling the nanostructure of 2D channels. Even though MXene flakes have emerged as potential building blocks for MXene/polymer membrane design, more rigorous research is still needed to investigate further their applications in wastewater treatment, solvent purification, and gas separation, among others.^[^ ^227^ ^]^ For the first time, Wu et al. used Ti_3_C_2_T*_x_* in a PEI or PDMS matrix to create TFN membranes supported by polyaniline (PAN).^[^ ^228^ ^]^ In isopropanol, the oligomeric (200 to 1000 Da) polyethylene glycol (PEG) molecules were effectively rejected by all membranes, regardless of the loading ratio of MXenes and the kind of polymer matrix. Furthermore, PAN/PEI‐Ti_3_C_2_T*_x_*_‐4_ (with 4% MXene loading) was able to reject PEG‐800 98.4% of the time (at 10 bar). Hao et al. used a similar strategy to investigate the impact of chemical functionalization on PEG rejection.^[^ ^229^ ^]^ Various solvents benefit from different functions when the MXene loading ratio is fixed at 3 wt%. Han et al. synthesized MXene membranes by integrating Ti_3_C_2_T*_x_* into a polyimide (P84) matrix by phase inversion (PI) and then crosslinking with triethylenetetramine (TE) (TETA).^[^ ^225^ ^]^ A large flux (268 L m^−2^ h^−1^) of gentian violet (408 g mol^−1^) at 0.1 MPa and ambient temperature was successfully rejected by adjusting the filling ratio. After crosslinking, their membrane demonstrated remarkable solvent resistance to DMF, acetone, and methanol. MXene‐based membranes, as previously discussed for dye removal from aqueous samples, are likewise effective in rejecting colors from organic solvents.

#### Advances in Desalination Techniques Using MXene and Its Polymer Hybrids for Water Remediation

Half of the world's population is expected to live in water‐stressed regions by 2025. Increasingly contaminated and limited water supplies need the development of alternate water sources. As interest grows in the use of brackish water with salinities ranging from 0.5% to 30%, seawater desalination (salinity of 35 g L^−1^ or 3.5%) remains the silver bullet of safe water. To be sure, desalination is linked to water energy since, although electricity generation may use water to some extent, creating clean water is a labor‐intensive process.^[^ ^231^ ^]^ Despite the widespread use of technologies like (RO) and thermal distillation, which require considerable amounts of energy and money, there is no method for removing salt ions from solution at temperatures close to the thermodynamic energy limit.^[^ ^232^ ^]^ Many systems employ membranes, and each has its own set of performance restrictions based on membrane rejection, stability, energy efficiency, and flow. Due to pre and post‐treatment procedures, RO membranes have theoretical efficiency close to the thermodynamic limit, but the total energy needed may be up to four times that amount.^[^ ^233^ ^]^ Electrochemical desalination through capacitive electrolysis is an increasingly popular alternative method. As compared to membrane‐based size‐exclusion separations, salt is separated from water using lesser energy in this technique.^[^ ^234^ ^]^ Due to the fact that the amount of energy needed for RO increases with the amount of water being processed, it is ideal for high‐salinity waters. Traditional CDI may be used for low‐salinity streams like brackish water since its energy consumption scales with the quantity of salt removed.^[^ ^235^ ^]^ Additionally, a variety of desalination membranes may benefit from MXenes. Using MXenes for various desalination tactics, this section intends to demonstrate that MXenes may soon provide a low‐energy saltwater desalination method to meet present and future water shortages.^[^ ^236^ ^]^ Desalination applications might benefit from the use of MXenes and MXene‐based membranes. A low contact angle of 21.5° and great stability in water even after vigorous shaking make MXene (Ti_3_C_2_T*_x_*) a suitable option for desalination applications.

**Figure 17:**
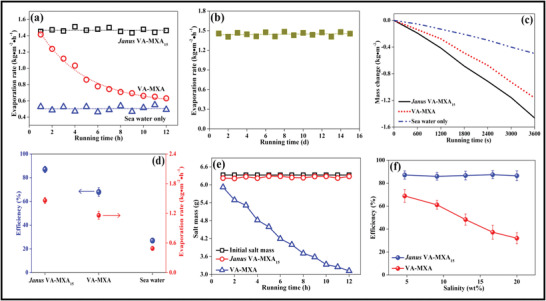
a) Comparison of evaporation rates for the Janus VA‐MXA_15_ and VA‐MXA within 12 h of irradiation. b) Water evaporation rates of the Janus VA‐MXA_15_ immersed in Yellow seawater for 15 d, 12 h each day. c) Mass change of Yellow seawater over time and d) water evaporation rates and corresponding energy conversion efficiencies of Janus VA‐MXA_15_, VA‐MXA, and without any absorber under 1 sun illumination. e) Variation of the salt mass of bulk water under the absorbers as a function of irradiation time. f) Influence of salt concentration on the steam generation efficiency of the Janus VA‐MXA_15_ and VA‐MXA. Reproduced with permission.^[^ ^163^ ^]^ Copyright 2019, American Chemical Society.

Using CDI, anions, and cations are separated and deposited on the anode and cathode, respectively, in a very energy‐efficient process. An optimal electrode in the CDI process requires a large surface area, superior electrical conductivity, and excellent stability.^[^ ^237^ ^]^ CDI electrode materials based on MXenes#x00027; improved pseudo‐capacitive characteristics have evolved. Due to its high electrical conductivity, hydrophilicity, and adjustable surface, the MXene‐based CDI electrode demonstrated a remarkable adsorption capacity for both cations and anions.^[^ ^238^ ^]^ MXenes‐derived membranes are an excellent candidate for use in solar desalination because of their high light‐to‐heat conversion efficiency. Furthermore, MXenes#x00027; capacity to evaporate water via photothermal evaporation is an energy‐efficient feature. An additional energy‐efficient feature of MXenes is their photothermal water evaporation.^[^ ^163^ ^]^ Two layers of VAMXA, hydrophilic at the bottom and hydrophobic at the top, were found to be effective in desalination, as reported by Zhang and co‐workers.^[^ ^163^ ^]^ Under Ar protection, a PTFE mold with a Titanium plate freezes MXene derived from the Ti_3_AlC_2_ phase. The resulting MXene is then removed from the mold and placed in a vacuum (**Figure** **17**). Vacuum freeze‐drying removes ice crystals from Ti_3_C_2_ nanosheets, resulting in a vertically aligned framework. In order to create the hydrophobic layer, the independent VA‐MXA was deposited in a hexagonal sponge mold and floated over fluorinated alkyl silane under a vacuum before drying under an Ar atmosphere.^[^ ^166^ ^]^

**Figure 18:**
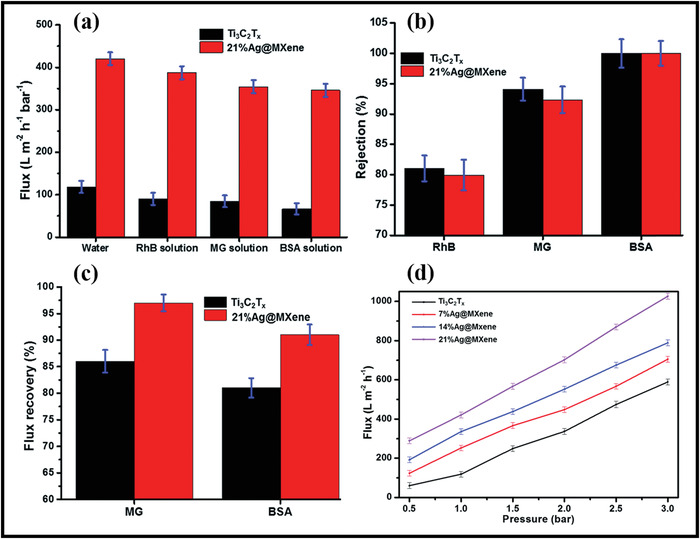
a) Comparison of flux recovery after organic fouling in MXene (Ti_3_C_2_T*_x_*) and 21%Ag@MXene membranes. Comparison of the performance of the MXene (Ti_3_C_2_T*_x_*) and 21%Ag@MXene membranes for the separation of RhB, MG and BSA molecules at 25 °C: b) Flux, and c) Rejection. d) Pure water flux of Ti_3_C_2_T*_x_* and Ag@MXene composite membranes. Reproduced with permission.^[^ ^244^ ^]^ Copyright 2018, Royal Society of Chemistry.

In pervaporation desalination, water diffuses across a membrane and then evaporates into the gaseous phase on the opposite side of the membrane, creating a brine solution.^[^ ^238^ ^]^ An unsupported MXene and PAN Polymeric substrates composite were used to vacuum filter the MXene suspension.^[^ ^239^ ^]^ To synthesize the MXene/PAN composite, a specified number of MXene nanosheets were placed on a PAN substrate.^[^ ^240^ ^]^ In contrast, independent MXene membranes were created by exfoliating MXene off substrates after drying for 24 h using polycarbonate (PC). The color of the MXene layer changes from light green to dark green as the number of MXene nanosheets rises. Independent or supported membranes, MXene nanosheets can be used to reject molecules and ions based on their size and charge preferences.^[^ ^241^ ^]^ Since of the surface functions of MXene flakes, synthetic MXenes membranes may be used for selective separations because they have a space between their interlayers. MXenes, on the other hand, are hydrophilic, and their termination ratio is highly dependent on the manner of production. As a result, MXene nanosheets are often preferred for large‐scale membrane production. Based on the hydration radius and ionic charges, MXene membranes have been designed for salt separation.^[^ ^224^ ^]^ Vacuum‐assisted filtering was used to coat the polymeric supports with a thin Ti_3_C_2_T*_z_* MXene coating, resulting in nanochannels with few meso‐ and macropores. MXene demonstrated 475% more water flux than GO membranes of the same thickness, which is equivalent to the ion sieving and water flux capabilities of these membranes. This rapid water penetration was attributed to the coexistence of water molecules between the layers under the moist environment, generating a route for water transport.^[^ ^70^ ^]^ Due to their differing physicochemical features, MXene and GO have different transport properties in their membranes. Cations with hydration radii larger than interlayer galleries, which can sieve single to triple‐charge metal and dye cations because of the negative surface charge of MXene adsorbing and repelling cations, were hindered by the MXene membranes, according to density functional theory (DFT) calculations.^[^ ^242^ ^]^ The different charges of the ions affect the intercalation energy barrier and sieving rates, which alter the electrostatic interactions. GO membranes could not separate multivalent metal ions as well as Ti_3_C_2_T*_z_*‐based membranes.^[^ ^242^ ^]^ This shows the potential of Ti_3_C_2_T*_z_*‐based membranes in real‐world applications. An external voltage may modulate the MXene‐based membranes#x00027; water flow and rejection rates. For example, membranes made of MXene have the ability to remove NaCl, methylene blue, and MgSO_4_. Rejection efficiency was reduced while water flow rose when a positive voltage was applied.^[^ ^226^ ^]^ Conversely, water permeance was reduced, but rejection efficiency was enhanced. These membranes show excellent water flow and stability. As a reducing agent and a separation layer, MXene may also alter the surface of MXene by reducing AgNO_3_ to Ag nanoparticles (NPs).^[^ ^243^ ^]^ In comparison to the nascent MXene membrane, the introduction of NPs boosted water penetration by roughly fourfold while maintaining rejection. In this case, the membranes may have increased the interlayer distance and formed a short transport channel owing to more vacancies, short water paths, and better hydrophilicity of the membranes. Ding et al. designed AAO supported Ti_3_C_2_T*_x_* MXene‐based 2D membrane.^[^ ^104^ ^]^ The water permeability of these membranes was measured and found to be as high as 1000 L m^−2^ h^−1^ bar^−1^. Membrane separation efficiency has also been examined by utilizing molecules of various molecular sizes. Rejection rates were more than 90% observed for molecules with diameters larger than 2.5 nm. Liu et al. designed a polyacrylonitrile‐supported Ti_3_C_2_T*_x_* membrane with 60 nm thickness for NaCl separation.^[^ ^244^ ^]^ At 65 °C, the as‐prepared membranes had water permeance of 85 L m^−2^ h^−1^ bar^−1^ with a 99.5% rejection rate.^[^ ^243^ ^]^ Ag@MXene membrane developed by Mahmoud and colleagues showed improved water permeance, rejection, and antifouling capabilities at a thickness of 470 nm. Under the same circumstances, these membranes had water permeance of 420 L m^−2^ h^−1^ bar^−1^, which was higher than the 118 L m^−2^ h^−1^ bar^−1^ of pure MXene membranes as shown in (**Figure** **18**). Additionally, these membranes are more stable in synthetic saltwater than other membranes. To separate monovalent ions, researchers Lu and co‐workers used self‐crosslinked MXene membranes (SCMMs).^[^ ^252^ ^]^ The self‐crosslinking reaction has been used to cross‐link the MXene nanosheets#x00027; terminal functional groups. Compared to the pure MXene membranes (16.6), this method provided regulated swelling characteristics (15.4) and outstanding stability up to 70 h. As a result of self‐crosslinking between MXene sheets, SCMMs had a penetration rate around two orders of magnitude lower than pure MXene membranes, indicating a better ions exclusion capability. According to Ma and colleagues, using MXene/P84 copolyimide membranes resulted in almost 100% dye removal.^[^ ^225^ ^]^ MXene membranes have been studied extensively, despite many studies having been published. However, MXene, like other 2D materials, tends to inflate into the water and delaminate quickly owing to electrostatic and hydrogen bonding, which in turn decreases the separation efficiency of membranes. To improve the separation capabilities of Ti_3_C_2_T*_x_* membranes, Wang and co‐workers developed pillared M‐SAT lamellar membranes in which metal cations like Ca^2+^ and Mn^+^ function as pillars between MXene nanosheets, which raised their diameters from 13.8 to 15.2 nm.^[^ ^246^ ^]^ The d‐spacing of 2D laminates has a substantial impact on their selectivity. High water permeance Na_2_SO_4_ salts were completely rejected by the ultrathin Mn‐SAT pillared membrane with better d‐spacing. When compared to other MXene‐based membranes, they demonstrated much‐reduced swelling. Ding et al. have created non‐swelling MXene‐based laminates with enhanced water purification separation capabilities.^[^ ^247^ ^]^ Al^3+^ ions have been incorporated into MXene nanosheets. Al^3+^ and oxygen functional groups on MXene formed strong electrostatic interactions, preventing membrane swelling in water and boosting stability for up to 400 h. A high rejection rate of 89.5–99.6% against NaCl was also found, as was excellent water permeance of 1.1–8.5 L m^−2^ h^−1^ bar^−1^. MXene‐based lamellar membranes for water filtration and desalination have received significant attention. Most importantly, laminates are created on polymeric supports for greater rejection and permeance.

#### Engineering Adsorption‐Based Water Remediation Techniques Utilizing MXene and Its Polymer Hybrid Membranes

In light of the present energy crisis, a variety of approaches have been employed to provide environmentally friendly fuel. Water‐splitting reactions have gained interest as a renewable energy source since solar energy is an ideal source of renewable energy.^[^ ^249^ ^]^ It is imperative that we use green energy harvesting systems to tackle the current crisis in fossil fuels. After being discovered recently, transition metal carbides and nitrides termed “MXenes” have gained researchers#x00027; attention.^[^ ^250^ ^]^ Because of their high conductivity and enormous specific surface area, they have demonstrated encouraging development in various applications, including catalysis, energy storage, and biomedicine.^[^ ^182^ ^]^ As a result, they have excellent adsorption and photocatalytic oxidation air pollution capabilities. More reactive sites for solvent reactions are exposed thanks to the 3D porous nano floral structure, and the diffusion length of photoinduced charge carriers is reduced according to their high specific surface area. A low‐cost and widely used photoredox technology, photocatalysis removes various organic contaminants from water while generating just CO_2_ and H_2_O_2_ as waste products.^[^ ^251^ ^]^ Because of this, several efforts have been made to use semiconductor photocatalysts in pollution degradation in an effort to attain practical applications.^[^ ^252^ ^]^ Since semiconductor heterojunction‐based catalysts seem to be the most productive and simple method for degrading contaminants in aqueous media, they have received the greatest attention.^[^ ^253^ ^]^ Many new heterojunction materials, including g‐C_3_N_4_/Ti_3_C_2_T*_x_* and Bi_2_WO_6_/Ti_3_C_2_T*_x_*, have recently been studied for photocatalytic degradation of organic contaminants. Using a 3D porous TiO_2_@Ti_3_C_2_ nanoflower heterojunction, Quyen and co‐workers could degrade RhB through photocatalysis.^[^ ^254^ ^]^ The fabricated photocatalyst removed 97% of RhB in 40 min of light irradiation, whereas pure TiO_2_ and Ti_3_C_2_ MXene could only remove 36% and 29% of the RhB, respectively, under the same circumstances. This innovative technique demonstrates that Nano flower porous structures of TiO_2_/Ti_3_C_2_ play an important role in improving the surface area, light‐harvesting, and charge separation for photocatalytic activity. Therefore, the unique properties of TiO_2_/Ti_3_C_2_ make it a potential photocatalyst without the use of noble metals. Mashtalir et al. observed adsorption of MB on Ti_3_C_2_T*_x_* MXene in the dark dramatically increased within the first 8 h due to the favorable electrostatic interaction of negatively charged MXenes and cationic dye of MB, whereas no changes in AB 80 adsorption even after 20 h was due to electrostatic repulsion of negatively charged MXene and anionic AB 80.^[^ ^182^ ^]^ Under 5 h of UV irradiation, AB 80 and MB were both photodegraded around 62% and 81%, respectively, by Ti_3_C_2_T*_x_*. In the first 2 h, the authors found evidence of an interaction between MXene and MB based on the use of XRD and XPS analysis, which showed that active adsorption, layered structure wedging, and/or chemical MXene transformation, as well as the oxidation of Ti_3_C_2_T*_x_*, led to titania formation and dissolved oxygen concentration increases. A recent study by Kim et al. found that the adsorption performance of MB (51.7%) and MO (34.9%) on Ti_3_C_2_T*_x_* MXenes combined with an ultrafiltration membrane (MXene‐UF) was lower than that of granular activated carbon‐UF (57.7 and 47.9% for MB and MO, respectively), while MXene‐UF exhibited higher selectivity for MB and MO (the normalized flux 1/4 0.92) than GAC‐UF (0.72 for MB and 0.75 for MO) due to electrostatic attraction between the membranes and dyes.^[^ ^255^ ^]^ MXene‐enhanced UF's dye retention at high pH and low background ions was also attributable to its more negative terminations and bridging action. The surface area of powder‐activated carbon (470 m^2^ g^−1^) was 50‐fold greater than that of MXene (10 m^2^ g^−1^), but MXene had a stronger elimination capacity because of its larger negative surface charge reported by Jun and co‐workers.^[^ ^256^ ^]^ As FTIR and XPS investigations indicated, ion exchange, electrostatic attraction, and the development of inner‐sphere complexes were the primary elimination mechanisms. Similarly, According to Peng et al., even at high concentrations, the 2D alk‐MXene (Ti_3_C_2_ (OH/ONa)*_x_* F_2‐_ *_x_*) material still prefers to remove Pb(II), and the equilibrium adsorption time was just 120 seconds.^[^ ^156^ ^]^ For Pb(II), Ca(II), and Mg(II), Gibb's free energy of hydration is 1425, 1505, and 1830 kJ mol^−1^, respectively. Due to Pb (II)’s low hydration energy, it was predicted that Pb(II) would be eliminated first, followed by Ca(II), and then Mg(II), which was confirmed by the experiments. Other MXene‐based membranes were discussed in **Table** **3**.

**Table 3:** MXene based Membranes for the removal of dye, and ions from wastewater

MXene‐based membranes	Synthesis method	Pollutants	Outcomes	Refs.
Ti_3_C_2_T*_x_*/CNT	Vacuum filtration	Dye, ion	MO and NaCl were adsorbed around 95.4% and 20.6%, respectively	[257]
Ti_3_C_2_T*_x_*/CA	Doctor blade	Dye	RhB and MG were adsorbed around 92 and 98%, respectively	[258]
Ti_3_C_2_T*_x_*/GO	Vacuum filtration	Dye	MnB was adsorbed around 94.6%	[251]
Ti_3_C_2_T*_x_* composite	Vacuum filtration	Dye, ion	CR and NaCl were adsorbed around 92.3% and 13.5%, respectively	[259]
Ti_3_C_2_T*_x_*/GO	Vacuum filtration	Dye	CG and NR were adsorbed around 95%	[260]
Tannic acid/Ti_3_C_2_T*_x_*	Vacuum filtration	Dye, ion	CR and MgSO_4_ were adsorbed around 96% and 13%, respectively	[261]
Ti_3_C_2_T*_x_*/GO	Vacuum filtration	Dye	MnB and MO were adsorbed around 98% and 96%, respectively	[262]
PA/Ti_3_C_2_T*_x_*	Interfacial polymerization	Ion	NaCl was adsorbed around 98%	[152]
Ti_3_C_2_T*_x_*/DGO	Vacuum filtration	Dye, ion	DR and NaCl were adsorbed around 98% and 9.6%, respectively	[263]
Ag/Ti_3_C_2_T*_x_*	Vacuum filtration	Dye	MG and RhB were adsorbed around 92% and 80%, respectively	[244]
Ti_3_C_2_T*_x_*/GO	Vacuum filtration	Dye, ion	MnB and NaCl were adsorbed around 99% and 11%, respectively	[251]
Ti_3_C_2_T*_x_*	Vacuum filtration	Ion	MgCl_2_ was adsorbed around 82%	[264]
Ti_3_C_2_T*_x_*	Vacuum filtration	Ion	NaCl and KCl were adsorbed around 99% and 97%, respectively	[265]
Ti_3_C_2_T*_x_*	Vacuum filtration	Ion	Na_2_SO_4_ was adsorbed completely	[247]
Ti_3_C_2_T*_x_*	Vacuum filtration	Dye	EB and Cyt. C were adsorbed around 90% and 93% respectively	[105]
Ti_3_C_2_‐TiO_2_	Multilayer MXene mixing with H_2_O_2_	Dye	RhB was adsorbed around 96%	[266]
Ti_3_C_2_/TiO_2_/CuO	Annealing	Dye	MO was adsorbed around 99%	[252]
BiOBr/Ti_3_C_2_	Hydrothermal method	Dye	RhB was adsorbed completely	[267]
TiO_2_@C derived from Ti2CT*_x_*	Hydrothermal	Dye	MB was adsorbed around 85%	[268]
La‐ and Mn codoped BiFeO_3_/Ti_3_C_2_	Sol‐gel	Dye	CR was adsorbed completely	[269]
Ti_2_C/TiO_2_, Ag_2_O, Ag, PdO, Pd, or Au	Sol‐gel	Organic contaminant	Salicylic acid was adsorbed around 95%	[270]
Ti_3_C_2_‐OH/ln_2_S_3_/CdS	Hydrothermal	Dye	EB and Cyt. C were adsorbed around 96% and 99% respectively	[271]
Ag_3_PO_4_/Ti_3_C_2_	Self‐assembly	Dye	MO was adsorbed completely	[272]
2D *α*‐Fe_2_O_3_ doped Ti_3_C_2_	Sol‐gel	Dye	RhB was adsorbed around 98%	[273]

### 5.3 Advances in Combating Radiation‐Based Contamination Utilizing Mxene and Its Polymer Hybrids

The radiation‐based electronic and nuclear contaminations are the most growing type of pollution, raising global concern to combat it. MXene and its hybrid based membranes owing to high specific surface areas and superior adsorption efficacies, have been anticipated as next‐generation radiation remediation materials.

#### Advancements in EMI Shielding Techniques Utilizing MXene and Its Polymer Hybrid Membranes

**Figure 19:**
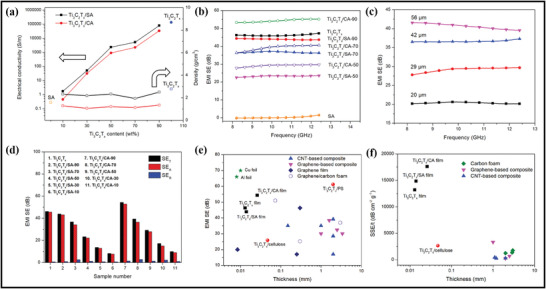
a) Electrical conductivity and density of Ti_3_C_2_T*_x_*, SA, Ti_3_C_2_T*_x_*/SA films, and Ti_3_C_2_T*_x_*/CA aerogel films (Ti_3_C_2_T*_x_* content in SA matrix varies from 10 to 90 wt%), b) EMI SE of SA, Ti_3_C_2_T*_x_*, Ti_3_C_2_T*_x_*/SA films, and Ti_3_C_2_T*_x_*/CA aerogel films, c) EMI SE of Ti_3_C_2_T*_x_*/CA‐50 aerogel film as a function of the sample thickness (µm). d) Comparison of EM shielding mechanism (SET, SEA, and SER) for each sample. e) EMI SE versus thickness of previously reported different materials. f) Comparison of the SSE/t as a function of the sample thickness (µm). Reproduced with permission.^[^ ^117^ ^]^ Copyright 2019, Wiley‐VCH.

Electromagnetic interference (EMI) has emerged as a new type of pollution due to the rapid growth of wireless transmission technologies, particularly in the high‐frequency range. In order to reduce electromagnetic radiation, EMI shields must have a reasonably high electrical conductivity, which is a key function of their inherent charge carriers and electromagnetic fields.^[^ ^274^ ^]^ Metal, conductive polymers, carbonaceous compounds, and metal oxides are only a few materials that have been used to block electromagnetic radiation thus far. Traditional metal‐based shielding materials offer high EMI shielding performance, but their low electromagnetic wave absorptions and sensitivity to corrosion in hostile environments have limited their utilization.^[^ ^275^, ^276^, ^277^ ^]^ Foam structures have recently attracted a lot of attention as a technique to lower the density of shielding materials because lightweight materials are essential for aeronautical applications.^[^ ^278^ ^]^ Thus, carbonaceous materials, such as reduced GO, carbon foam, multiwall carbon nanotubes, and carbon black, have been widely used to manufacture EMI shielding materials. Several applications require millimeter‐scale thickness for carbon–polymer composite EMI shielding materials, limiting their use in many sectors.^[^ ^279^, ^280^ ^]^ Commercial EMI shielding needs (>30 dB) necessitate substantial membranes, but few EMI shielding materials can offer ultrathin thickness, high flexibility, and outstanding EMI SE characteristics at the same time.^[^ ^281^ ^]^ EMI shielding materials based on 2D MXenes, which have a large specific surface area, strong electrical conductivity, and lightweight characteristics, have been proven to be the most effective.^[^ ^148^ ^]^ Regarding EMI shielding, MXene–polymer membranes have shown high conductivities even at modest MXene loadings, as previously indicated. Multiple internal reflections of electromagnetic radiation within the membrane are provided by multilayer structures, significantly improving EMI shielding. As a result, the MXene–polymer membrane quickly becomes one of the most important EMI shielding materials due to its low percolation threshold, exceptional mechanical features, and improved shielding efficacy.^[^ ^282^ ^]^ In a recent study, Zhou and co‐workers found that cross‐linking SA molecules with calcium ions improved the EMI shielding of the Ti_3_C_2_/SA membrane.^[^ ^117^ ^]^ After injecting the Ti_3_C_2_/SA composition into the CaCl_2_ solution, the unbound alginate particles developed a complex with the Ti_3_C_2_‐adsorbed alginate. VAF and freeze‐drying methods yielded Ti_3_C_2_/calcium alginate (CA) aerogel films with varied MXene loadings with high specific EMI shielding performance (17 586 dB cm^2^ g^−1^). The sponge‐like structure benefits the Ti_3_C_2_/CA aerogel membrane, which has a greater EMI shielding performance (54.3 dB at a thickness of 26 m) than pure Ti_3_C_2_ and Ti_3_C_2_/SA films. While MXene concentration increases, the tensile strength and fracture strain drop simultaneously, as shown in **Figure** **19**a–f. The highest mechanical capabilities (tensile strength of 154 MPa and strain of 16.7%) are attained when MXene content is 50 wt%. Additionally, the enhanced conductivity of the Ti_3_C_2_/CNF membrane yielded excellent specific EMI shielding performance. With an increase in MXene concentration and a rise in frequency, the particular EMI shielding efficacy of the Ti_3_C_2_/CNF membrane improves. With an increase in MXene concentration and a rise in frequency, the particular EMI shielding efficacy of the Ti_3_C_2_/CNF membrane improves. At 12.4 GHz, a maximum of (2647 dB) is achieved using 90 wt% Ti_3_C_2_ flakes.^[^ ^168^ ^]^

Biocompatible sodium alginate (SA) and numerous MXene materials, including Ti_3_C_2_, Mo_2_, TiC_2_, and Mo_2_Ti_2_C_3_, were used by Shahzad et al. to produce the MXene/polymer membranes.^[^ ^115^ ^]^ After analyzing six membranes of varied thicknesses, the maximum EMI shielding efficacy (92 dB) was obtained with a 45 m thick membrane. Ti_3_C_2_T*_x_*/SA membrane's EMI shielding efficacy increased with increasing MXene concentration and decreased with increasing frequency. At 8.2 GHz, the 90 wt% Ti_3_C_2_T*_x_*/SA membrane attained a maximum of 57 dB. Liu and colleagues synthesized Ti_3_C_2_/PEDOT: PSS using a novel VAF technique to evaluate the impact of MXene loading on the EMI shielding capability.^[^ ^190^ ^]^ At 82.5 wt% MXene loading with a thickness of 11 m, 42.1 dB specific EMI shielding efficiency was shown. At the same time, superior EMI shielding efficiency of 19 497.8 dB was also attained by Ti_3_C_2_/PEDOT: PSS membrane at an increasing percentage of MXene. In addition, the composite membranes containing PPy and PANI were tested for EMI shielding efficacy. **Table** **4** demonstrates the EMI shielding using various MXenes membranes. Furthermore, MXene–polymer membranes with various industrial polymers demonstrated EMI shielding, such as Ti_3_C_2_/natural rubber, Ti_3_C_2_/epoxy, Ti_3_C_2_/polystyrene, Ti_3_C_2_/polyimide, etc., has been extensively studied.^[^ ^282^ ^]^ Graphene oxide and carbon nanotubes were also added to the MXene–polymer membranes to enhance their conductivities.^[^ ^283^ ^]^ The high‐performance EMI shielding material attributes often include high conductivity, ease of manufacturing, cheap cost, lightweight, and higher strength.^[^ ^284^ ^]^ Electromagnetic shielding using MXene–polymer membranes packed with conductive MXene flakes has been widely used in flexible robot joints, wearable gadgets, and weaponry due to its great processability and low density.^[^ ^285^ ^]^ Reflection or absorption of electromagnetic radiation may be used to shelter a device from EMI while low energy levels are communicated.^[^ ^286^ ^]^ On the other hand, reflection‐dominated EMI shielding materials emit twice as much pollution as absorption‐dominated EMI shielding materials.^[^ ^287^ ^]^ A lightweight Ti_2_C_1_/PVA composite foam and a Ti_2_C/PVA film were recently developed by Xu et al. and revealed exceptional specific shielding efficacy (0.15% MXene loading) of 4770 dB cm^2^ g^−1^ for the Ti_2_C/PVA film and 5136 dB cm^2^ g^−1^ for the Ti_2_C/PVA foam.^[^ ^288^ ^]^ Both Ti_2_C and PVA composites#x00027; electromagnetic wave reflection and absorption were thoroughly studied. Interestingly, the absorption/reflection ratio of Ti_2_C/PVA foam (13) is significantly higher than that of Ti_2_C/PVA films (0.91 and 0.86), indicating the EMI shielding of Ti_2_C/PVA foam is dominated by absorption rather than reflection. Furthermore, the increased absorption effectiveness is largely due to the foam's dipole polarization and interfacial polarization, the latter of which gives fresh inspiration for shielding materials that rely heavily on absorption.

**Table 4:** EMI shielding performances of MXene/polymer membranes

MXene/polymer	Method	*t* [µm]	SE(dB@GHz) & SE/t [dB µm^−1^]	wt [%]	Range [GHz]	*C* [s m^−1^]	Refs.
Ti_3_C_2_/epoxy	HP	1400	17@14.6 0.008	50	12.4–18.0	–	[282]
Ti_3_C_2_/wax	CP	1000	76.1 0.076	90	8.2−12.4	0.9	[280]
Ti_3_C_2_−CNT/paraffin	CP	1550	52.9@7.15 0.034	35	2.0−18.0	–	[281]
Ti_3_C_2_/PVB/Co_2_Z	DC	2800	46.3@5.8 0.017	10	8.2−12.4	–	[290]
Ti_3_C_2_/PEDOT: PSS	VAF	11.1	42.1@8.2 3.793	87.49	8.2−12.4	34 052	[191]
ZnO‐Ti_3_C_2_/paraffin	HP	4000	26.3@17.4 0.007	25	10.0−18.0	–	[148]
Ti_3_C_2_/PS	HP	2000	62@12.4 0.031	6.2	8.2−12.4	1081	[291]
Ti_3_C_2_/CNF	VAF	47	24@8.2 0.511	90	8.2−12.4	739.4	[168]
Ti_3_C_2_/Ni_0.5_Zn_0.5_Fe_2_O_4_/paraffin	CP	6500	42.5@13.5 0.007	5	0.2−18.0	–	[292]
Ti_3_C_2_/CA‐aerogel	VAF	26	54.3@8.2 2.088	90	8.2−12.4	33 832	[117]
Ti_3_C_2_/CNF	VAF	100	42.7@≈7.0 0.427	–	2.0−18.0	4360	[120]
Ti_3_C_2_/PPy/PET	DIP	1300	90 0.069	‐	8.2−12.4	1000	[147]
Ti_2_C/PVA‐film	PR	100	26	7.6	8.2−12.4	8.5 × 10^−5^	[290]
Ti_3_C_2_/PVA/MWCNT/PSS	LBL	0.17	2.81 16.529	4.2	8.2−12.4	13 000	[130]
Ti_3_C_2_/PANI/paraffin	CP	1800	56.3@13.8 0.031	26.5	2.0−18.0	–	[293]
Ti_3_C_2_‐rGO/epoxy	HP	2000	56.4@12.4 0.028	0.74	8.2−12.4	695.9	[284]
Ti_3_C_2_/NR	VAF	251	53.6@12.4 0.214	6.71	8.2−12.4	1400	[107]
A‐Ti_3_C_2_/epoxy	DC	2000	41@8.2 0.021	15	8.2−12.4	105	[139]
Ti_3_C_2_/PI‐aerogel	DC‐FD	3000	45.4@9.59 0.015	0.084	1.0−18.0	4.0	[288]
Ti_3_C_2_/PPy/paraffin	CP	3200	49.2@8.5 0.015	25	2.0−18.0	–	[294]

#### Advances in Nuclear Radiation Shielding Efficacies of MXenes and Its Polymer Hybrid Membranes

Traditional fossil fuels are becoming increasingly rare, and nuclear power has emerged as a solution to this challenge.^[^ ^295^ ^]^ Nuclear energy development will unavoidably emit large quantities of radionuclides with long half‐lives and great mobility into the aquatic environment, such as strontium (_90_Sr), barium (_133, 140_Ba), cesium (_137_Cs), thorium (_232_Th), and uranium (_235, 238_U). These radionuclides are dangerous to humans and the environment at low concentrations because of their high chemical toxicity and long‐term radiological effects.^[^ ^296^ ^]^ Therefore, there has been considerable interest in and study of MXene‐membranes having 2D layered structures in the domains of nuclear waste management and pollution remediation in recent years. Wang et al. discovered the multilayered V_2_CT*_x_* nanomaterial was an effective adsorbent for the removal of U(VI) owing to its high adsorption capacity (174 mg/g), good selectivity, and quick reaction kinetics.^[^ ^297^ ^]^ Experiments using DFT and EXAFS showed that U (VI) was adsorbed on the vanadium‐based MXene through ion exchange and bidentate inner‐sphere complexation. To remove radionuclides from Ti_3_C_2_T*_x_*, the interlamellar space (2 nm) was very small, preventing the admission of U(VI) with a large radius. Ti_3_C_2_T*_x_’*s interlamellar space may be easily increased by synthesizing and keeping the original and modified MXenes under hydrated conditions, as recently reported by Wang et al. Ti_3_C_2_T*_x_*’s minimum enlarged c lattice parameter was increased from 1.52 to 20.18 after hydration and intercalation. The Ti_3_C_2_T*_x_*‐DMSO‐hydrated material has the highest absorption capacity to U (VI) (160 mg g^−1^) compared to Ti_3_C_2_T*_x_*‐dry (26 mg g^−1^), which is almost six times more.

## 6 Cutting‐Edge Future Prospects, Limitations and Alternate Solutions Associated With Mxene and Its Polymer Hybrid Membranes

The research on MXene‐based membranes has risen exponentially in the past two years. However, we feel that the majority of this development is based on applications that GFMs or other 2DMs may also meet (depending on the cases). In addition, most published investigations use MXene‐based membranes made exclusively by vacuum‐filtration of MXenes. This information suggests it would be more efficient to conduct a study in various steps: 1) centered on applications where MXenes can offer advantages over all the other 2DMs, and 2) investigating MXene–polymer nanocomposite in greater depth by looking at more existing and expandable techniques used during polymer‐based separation and purification. To go a step further, we think modeling/ simulation tools may be able to identify the regions and membrane patterns where MXenes could demonstrate excellent potential. Therefore, there were four Future Prospects: 1) promising membrane operations, 2) underused membrane formats, 3) simulation/modeling techniques, and 4) scale‐up activities included in the Future Prospects.

### 6.1 Capable Membrane Strategies Based on Mxene and Its Polymer Hybrids Devising Mxene and Its Hybrid‐Based Electro‐Responsive Membranes

In previous investigations on several material systems, electroresponsive membranes were proven to be promising for wastewater treatment. Using MXenes as electro‐responsive membranes is a good idea because of their excellent chemical, mechanical, and electrical properties.^[^ ^298^, ^299^ ^]^ In their groundbreaking work, Ren et al. demonstrated the usefulness of MXenes for fabricating electro‐responsive membranes.^[^ ^227^ ^]^ A vacuum‐assisted filtering process was used to deposit the MXene (Ti_3_C_3_T*_x_*) based membranes onto PVDF support. The surface charge of the MXene was then modulated in response to the external voltage, resulting in better rejection of metallic ions and a charged natural dye. They observed that a negative potential (0.6 V) restrains the penetration of divalent (MgCl_2_, Mg^2+^) cations and monovalent (NaCl, Na^+^) via MXene membranes under an osmotically driven state (i.e., concentration gradient). An increase in ion penetration rates may be achieved by increasing the positive potential to +0.4 V. MXene membrane might be negatively charged under vacuum, enhancing the rejection of a positively charged dye (methylene blue). A less intensive exfoliation approach yields bigger MXenes flakes, which have been shown to have higher electrical conductivity and mechanical stability, resulting in greater solute rejection. The degradation of the electro‐responsive capability of such MXene‐based membranes after numerous usage cycles would be interesting to investigate further. In addition, the electrochemical processes that occur throughout these procedures must be better understood. MXenes#x00027; electrochemical properties are of interest among researchers since they may be used in a broad variety of applications.^[^ ^193^, ^300^ ^]^ Further research into the interactions between ions and MXenes may open up new possibilities for MXenes‐based membranes in applications other than separations, as will this. The list of possible uses for this technology includes, but is not limited to, light‐controlled nanofluidic,^[^ ^238^ ^]^ osmotic energy harvesting, lithium–sulfur batteries, and fuel cells.^[^ ^101^, ^102^, ^110^, ^301^, ^302^ ^]^ On the other hand, advancements in these areas may aid in developing MXene‐based membranes for separation applications sooner rather than later. Because of this, we advise researchers to take a more comprehensive look at the applications provided by 2DMs like MXenes.^[^ ^303^ ^]^

### Architecting MXene and Its Hybrid‐Based Reactive Membranes

In situ elimination of organic or ionic pollutants through reductive and/or oxidative processes is a promising method for wastewater treatment based on nanomaterial‐enabled reactive membranes. With the reactive membranes, it may be possible to eliminate the need for extra separation procedures or the use of costly chemicals altogether.^[^ ^304^ ^]^ A high and long‐lasting catalytic activity is required from nanomaterials. Xie and co‐workers recently proved the possibility of such a strategy by eliminating Cr^4+^ (at neutral pH) using an MXene‐based membrane consisting of Ti_3_C_2_T*_x_* and GO nanosheets.^[^ ^305^ ^]^ Harmful (Cr^4+^) had previously been converted towards less hazardous (Cr^3+^) using Ti_3_C_2_T*_x_* nanosheets, as shown by the work of Ying and co‐workers.^[^ ^306^ ^]^ Furthermore, the Cr^3+^ ions were removed without additional treatment (at pH 5) to fulfill the drinking water requirement. Reactive membranes capable of transforming carcinogenic bromate (BrO_3_^−^) ions into less harmful bromide (Br^−^) ions have recently been shown by Pandey et al.^[^ ^307^ ^]^ They have obtained a remarkable reduction capacity of 321.8 mg BrO_3_^−^ per g of Ti_3_C_2_T*_x_* with a nearly full reduction without adding energy or catalyst (at pH 7). However, MXene (Ti_3_C_2_T*_x_*) was partially oxidized in the shown system, limiting its usefulness as a reactive membrane. The stability of MXenes must be improved to provide long‐term performance. Additionally, it is important to go beyond the bromate detoxifying uses in the near future. Bisphenol A (BPA), an endocrine disrupting chemical, has been degraded by an MXene‐based catalyst in recent research.^[^ ^308^ ^]^ Because of this, we think membranes built on MXene might be useful in detoxifying a broad spectrum of pollutants.^[^ ^309^, ^310^ ^]^ In addition, MXenes and their hybrids have been shown to be excellent (and diverse) electrocatalysts, frequently with remarkable performance, as initially proven for energy applications. MXenes, in our opinion, have the potential to develop “electro‐reactive” membranes as well.

### 6.2 Underutilized Membrane Formats Based on MXene and Hybrid Membranes

Both phase inversion (PI) and interfacial polymerization (IP) have not been extensively used to make MXene‐based membranes yet, which may change in the near future. In the following sections, we provide some excellent practices and lessons learned from MXene‐based membranes to facilitate the investigation of PI and IP for MXene‐based membrane research. For creating MXene‐based membranes, we also provide a short overview of layer‐by‐layer construction.

#### Phase Inversion Membrane Utilizing MXene and Hybrid Membranes

Specifically, we feel that membrane or TFN‐prepared polymer–MXene composites have the most promise for practical applications. PI is required for the production of both MMMs and TFNs. Only Han et al. have reported on the use of PI in the fabrication of MXene–polymer composite membranes, to the best of our knowledge.^[^ ^226^ ^]^ According to our research, there is still more to learn about the polymer's involvement, the fabrication processes#x00027; influence, and the role of MXenes in the PI process itself. Therefore, the phase separation behavior and the polymer chain packing are inextricably linked when performing PI with MXenes. When a solvent and coagulant (or nonsolvent) counter‐diffuse in polymer–solvent coagulant tertiary systems, hydrophilic additives have been demonstrated to aid the demixing process.^[^ ^311^, ^312^ ^]^ Polymer chains harden swiftly and with limited rearrangement capability during GFM‐polymer phase inversion, resulting in membranes with high surface size and pore density as well as high general porosity. For this reason, we believe MXenes have great potential for producing extremely permeable membranes, given their hydrophilicity and topological resemblance to GFMs.^[^ ^313^ ^]^ When MXenes are separated, they will be exposed to the membrane surface, which might lead to new anti‐biofouling membranes. Another consideration is that the concentration of MXenes in these systems must be adjusted since thermodynamic and rheological parameters govern the phase separation process.^[^ ^314^ ^]^ Due to the increased viscosity of the polymer dope solutions, excessive usage of MXenes might postpone demixing and densifying membrane structures. To get the most out of MXenes in PI‐based membrane design, it is critical to strike a balance between these two factors.

#### Interfacial Polymerization

A thick polymeric layer is formed when two highly reactive monomers react at the interface of two immiscible liquids (usually polyamide‐based). PA‐based TFC membranes manufactured by IP are of special interest for a broad spectrum of industrial separation processes. Improved chlorine resistance and decreased fouling propensity are two of the most sought‐after properties in IP‐based PA membranes.^[^ ^315^ ^]^ It has been demonstrated that integrating GO into PA membranes during IP may accomplish all four of these connected characteristics.^[^ ^316^ ^]^ Softer PA layers have been achieved by slowing down the migration of water‐based monomers inside an organic solvent. With their 2D shape, we predict MXenes to have the same impact. We also anticipate MXenes to successfully suppress chloride ions from replacing amidic hydrogen and halting chlorination because of their ability to create strong hydrogen bonds. MXenes, like GO, are both antibacterial and hydrophilic, making them ideal for preventing (bio)fouling.^[^ ^123^ ^]^ Because of this, we believe MXenes have the potential to be used in the construction of IP‐based membranes. Various research groups have previously developed PA‐based selective layers on different support membranes by crosslinking MXene‐incorporated PEI with TMC. It is possible to use this simple IP approach to fabricate TFN membranes. A thorough adjustment of numerous parameters is required while building IP‐based TFN membranes.

#### Layer‐by‐Layer Assembly

Compared to IP and PI, the layer‐by‐layer assembly method looks more efficient for membrane design.^[^ ^317^ ^]^ In the past few years, various layer‐by‐layer assembled film compositions have shown promising performance as separation membranes. Membranes based on layer‐by‐layer generally use polymeric components. Layer‐by‐layer membrane designs based on or including nanosheets are also popular since they combine the benefits of layer‐by‐layer material design with 2DMs.^[^ ^318^, ^319^ ^]^ MXene‐incorporated layer‐by‐layer films, on the other hand, have been created for applications such as super‐capacitive power storage and EMI shielding. MXene‐based membranes are expected to be effective in separation and purification applications. There are several ways to do the layer‐by‐layer method, including spinning and dipping as well as spraying or a mix of them.^[^ ^128^, ^130^ ^]^ In addition, the quality of films that are produced might vary considerably. In addition, the molecular characteristics of these films may be fine‐tuned by experimenting with the formation circumstances. As a result, we believe the layer‐by‐layer technique is a potential approach for building MXenes‐based separation membranes.

### 6.3 Simulation/Modeling Approaches for Architecting MXene and Its Hybrid Membranes

Experimenters have difficulty matching the appropriate material to the appropriate application because of the rapid growth of planar materials toolset, which also applies to MXenes. In the near future, computer‐aided materials design (CAM‐CAD) will have enormous potential for improving research outcomes. MXenes and their parent materials, MAX phases, show minute details of structure‐property interactions that are generally unavailable or difficult to examine experimentally via computational/theoretical analyses. Density functional theory ab initio calculations have accounted for the vast bulk of computational investigations to date (DFT).^[^ ^59^, ^60^, ^320^ ^]^ Structure, electrical, mechanical, magnetic, and electrochemical characteristics of MXenes, as well as chemical bonding and their relative stability, were all investigated in depth in the basic domain. Energy storage was the primary emphasis on the application side of the equation. The electrochemical properties of several MXenes are also particularly intriguing for membrane applications, as we mentioned before.^[^ ^243^ ^]^ A few pioneering simulation/modeling studies specifically concentrate on MXene‐based separation membranes that we would like to describe first. For example, Kurtoglu and co‐workers have shown MXenes like Ti_4_C_3_, Ta_3_C_2_, Ta_2_C, Ti_3_C_2_, and Ti_2_C*_n_* early work to have high metal content and stiffness.^[^ ^59^ ^]^ Anasori et al. tested MXenes with two distinct transition metals in this context (Cr, Ta, Ti, Nb, V, or Mo). In the form of M'M"C_2_ and M'M"C_3_, the outer and inner layer metals were the M′ and the M″ respectively.^[^ ^60^ ^]^ In contrast to Ti_3_C_2_T*_x_*, the electrochemical behavior of Mo_2_TiC_2_ is wholly distinct. Notably, the researchers have also provided experimental evidence to back up their predictions.

With DFT simulations, Berdiyorov and co‐workers have studied the water desalination potential of MXenes.^[^ ^243^ ^]^ To further understand how charge‐selectivity works, they looked at ionic transport via Ti_3_C_2_(OH)_2_. As the charge of intercalating ions changes, the distance between the MXene layers expands or shrinks dynamically, affecting ionic transport. MXene‐based ion‐selective membranes may be enhanced by modifying surface terminations, which vary surface charges. Using MD simulations in the future, porous MXenes#x00027; potential for membrane production may be further assessed. Even at its thinnest, MXenes are stacked nanosheets by nature. MXene nanosheets may not necessarily have in‐plane porosity as a consequence of point surface flaws. However, porous MXenes are possible and have previously been used in a variety of applications.^[^ ^321^, ^322^ ^]^ We thus recommend MD simulations as a critical ingredient for future research to better understand the transport behavior of MXene‐based membranes and maybe design superior membranes that employ porous MXenes.

### 6.4 Scale‐Up Potential of MXene and Its Hybrid‐Based Membranes

MXenes, as well as inherently MXene‐based membranes, are still in their infancy when it comes to predicting their economic viability, regardless of their performance advantages.^[^ ^323^ ^]^ In spite of extensive attempts to produce common MAX phases in large numbers, the vast majority of MAX phases are not yet commercially accessible at a reasonable price.^[^ ^324^ ^]^ However, since fluorine‐based etching chemistries have inherent safety problems, scaling up the manufacturing of MXenes is considerably more difficult.^[^ ^325^ ^]^ An approach that could be safer and simpler to scale is using strong bases as etchants. There are still improvements to be made in non‐fluorine‐based techniques for yield and product quality. MXene compounds with diverse surface chemical compositions may be produced using different etching techniques. This might be an opportunity to engineer the characteristics of membranes from the standpoint of membrane design. On the other hand, it's critical to investigate methods for turning MXenes into membranes that can be scaled up.^[^ ^207^, ^326^ ^]^ Technologically acceptable procedures, such as blade‐casting variants, are expected to be among the top options for both MMMs and NLMs. PI and IP approaches are also worth a thorough examination in this respect, as mentioned in detail above.

### 6.5 Intelligent MXene and Polymer Hybrid Membranes: Future Technology

**Figure 20:**
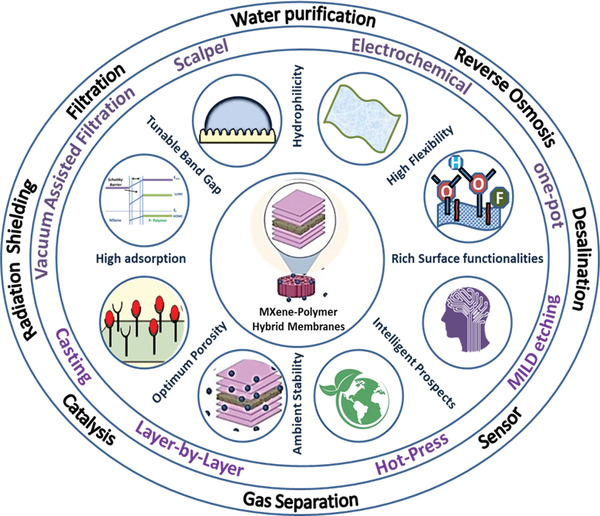
MXene–polymer hybrid membranes as future environmental remediation technologies.

MXene and its hybrids membrane especially architected with polymer hybrids are flexible in nature, which can be utilized to device future‐generation environmental remediation strategies. The current era of technological advancements has also witnessed the research and development of internet‐of‐things (IoTs), artificial intelligence (AI), machine learning, cloud computing, and 5G communications. Integrating these advanced membranes with IoTs, AI, 5G, and cloud computing can lead to the development of smart and intelligent environmental remediation technologies. Integrating IoT and 5G with these membranes allows remote access and real monitoring of the air, water, and radiation contamination and enables point‐of‐care analysis. Moreover, ML and AI can be incorporated into pretrained modules to devise point‐of‐solution module‐based membranes. Smart and intelligent membranes specifically designed for a particular contaminant can act based on pre‐trained algorithms for detection and provide the solution accordingly. For instance, a membrane devised for carbon dioxide detection and adsorption can be automated using modern age data analytics and rapid communication systems. On successful detection of CO_2_, the membrane can be used to adsorb or dissociate it accordingly. Similarly, a network of intelligent membranes can be installed along the water bodies for real‐time and remote monitoring of water contamination and its prevention, reducing human resources. Furthermore, AI and machine learning can be used to evaluate the adsorption, sensing, catalysis, and remediation efficacies of the membranes with varying stoichiometry and precursor's nature and concentration without undergoing actual experimentation. It reduces the secondary contamination, cost, energy requirements, and human efforts and resources to develop membrane‐based remediation technologies (**Figure** **20**).

## 7 Conclusion

The last decade has witnessed unprecedented research and growth in MXene and its hybrid‐based membrane technologies for environmental remediation. Especially, MXene–polymer hybrids owing to their abundant stoichiometry, stability, high specific surface area, flexibility, and tunable surface chemistries, have been utilized to device next‐generation membrane‐based environmental remediation. It is attributed to the synergistic effects and formation of interfacial heterojunctions among the organic‐inorganic precursors. MXene–polymer hybrid membranes have been extensively evaluated for air, water, and radiation remediation. The performance of MXene–polymer hybrid membranes as remediation technologies depends upon its fundamentals efficacies, including adsorption, catalytic and sensing behavior. These efficacies can be tuned by varying the stoichiometry, composition, and nature of precursors involved in membrane fabrication. Moreover, selectiveness and stability are the two key aspects of membrane‐based remediation technologies, which can be achieved by engineering the surface of membranes and their physicochemical attributes. However, the commercial viability and industrial applicability of MXene–polymer membrane are yet to be discovered. It is restricted by fundamental bottlenecks associated with fabrication, optimization, and storage. For architecting MXene–polymer hybrid membranes, the fabrication of MXene is a prerequisite and is the first processing step. The research is majorly dedicated to acid‐based etching strategies, especially HF or in situ HF based, which is highly corrosive in nature, responsible for environmental contamination, and raises serious health concerns for working researchers. Moreover, the reported scalable manufacturing of MXenes depends on a similar acid‐based approach. On the other hand, the mild‐acid‐based etching route (LiF‐HCl) is better than the HF route, resulting in fewer defects. However, the generation of extra cations or water among its layers is anticipated to influence its intrinsic properties. It indicates the requirement of bio‐derived, green, or mild bottom‐up strategies for the scalable fabrication of MXenes. Furthermore, the processing of MXene–polymer membrane architecture requires the optimization of precursors during the fabrication process. There is a requirement for simultaneous optimization of mechanical flexibility due to polymers and physicochemical attributes of MXenes for devising MXene–polymer hybrid membranes. Moreover, the research is primarily dedicated to Ti3C2T*_x_* for environmental remediation applications, and other stoichiometric compositions are yet to be explored. For instance, vanadium‐based MXene can be employed for selective detection of carbon‐based contamination and is better in terms of cytotoxicity. There is a fundamental issue of Ti_3_C_2_T*_x_* stability, which raises the devising of advanced storage strategies for industrial and commercial prospects. The major alternate solution is a post‐surface treatment of MXene flakes before processing MXene–polymer hybrid membranes. It can be done by utilizing the tunable abundant surface chemistries and physicochemical attributes of MXene flakes. These surface modifications not only contribute to MXene stability, prevent restacking, and enhance physicochemical attributes but also surge the interaction between polymer and MXene for a superior hybrid system. Hence, the life span, stability, and remediation efficacies like adsorption, catalysis, and sensing of MXene–polymer hybrids can be enhanced by surface modifications and tuning the compositions of hybrids. It can lead to scalable and industrial prospects of MXene–polymer hybrid membranes for devising environmental remediation strategies. Moreover, with the integration of modern day technologies like IoTs, AI, machine learning, cloud computing, and 5G communications, MXene–polymer membrane prospects to be next‐generation membrane‐based intelligent environmental technologies to shape smart and connected societies and cities.

## Conflict of Interest

The authors declare no conflict of interest.
